# Low-normal immunoglobulin suggests monoclonal B-cell lymphocytosis and constitutional CLL susceptibility: a dual-mechanism analysis

**DOI:** 10.1038/s41375-026-02987-2

**Published:** 2026-06-10

**Authors:** Ramon Cohen, Shay Nemet, Shira Bezalel-Rosenberg, Ilan Asher, Keren Mahlab-Guri, Pia Raanani, Daniel Elbirt, Liron Hofstetter

**Affiliations:** 1 Department of Internal Medicine B, Kaplan Medical Center, Clalit and Faculty of Medicine, Rehovot, Israel; 2 Department of Clinical Immunology, Allergy and AIDS, Kaplan Medical Center, Clalit, Rehovot, Israel; 3 Faculty of Medicine, Hebrew University of Jerusalem, Jerusalem, Israel; 4 Institute of Hematology, Davidoff Cancer Centre, Rabin Medical Centre, Petah Tikva, Israel; 5 The Gray Faculty of Medical and Health Sciences, Tel Aviv University, Tel Aviv, Israel; 6 Felsentein Medical Research Center, Rabin Medical Center, Petah Tikva, Israel

**Keywords:** Chronic lymphocytic leukaemia, Risk factors, Adaptive immunity

## Abstract

Low-normal serum immunoglobulin (Ig) levels may reflect early immune dysregulation preceding chronic lymphocytic leukemia (CLL), but their prognostic significance years before diagnosis is unclear. We conducted a large retrospective cohort study, including 294,712 adults aged 40–80 years with routine Ig testing and up to 10 years of follow-up. Immunoglobulin G (IgG), A (IgA), and M (IgM) levels were assessed a decade before diagnosis and dichotomized at the median values observed in individuals who later developed CLL. Multivariable Cox regression was performed, with sensitivity analyses stratified by baseline lymphocyte counts to distinguish constitutional effects from occult monoclonal B-cell lymphocytosis (MBL). Low-normal IgA ( < 187 mg/dL) demonstrated the strongest association with future CLL (adjusted hazard ratio [aHR] 2.62), followed by IgM (aHR 1.77) and IgG (aHR 1.43). In individuals with low-normal lymphocyte counts, IgM and IgG lost predictive value, whereas IgA remained independently associated with CLL risk. Dose-response analyses revealed steep monotonic gradients across the normal range. Temporal analyses demonstrated that IgA-associated risk extended beyond a decade prior to diagnosis, while IgM and IgG associations attenuated earlier. These findings support a dual-mechanism model in which low IgM and IgG reflect tumor burden from undiagnosed MBL, whereas low IgA identifies a constitutional susceptibility phenotype.

## Introduction

Chronic lymphocytic leukemia (CLL) is the most prevalent adult leukemia in Western populations, characterized by clonal expansion of mature B lymphocytes [1]. Despite advances in molecular and microenvironment understanding, the early immunological changes that precede clinical disease remain poorly defined. Hypogammaglobulinemia is common at CLL diagnosis [2, 3]. An earlier study showed up to 87% reduction under the normal range in at least one immunoglobulin (Ig) class [4]. at diagnosis. Low IgA (under the normal range) can be found around 30% at CLL diagnosis [5]. The association of Ig levels within the low-normal range years prior to diagnosis remains unclear.

Monoclonal B-cell lymphocytosis (MBL) is present in approximately 6-13% of individuals over age 40 and progresses to clinical CLL at a rate of 1–2% per year [6, 7]. Recent evidence demonstrates that MBL may be present decades before CLL diagnosis [8]. Despite high prevalence, most MBL remains undiagnosed absent systematic flow cytometry screening. The clinical utility of identifying asymptomatic MBL remains controversial, though knowledge allows personalized infection monitoring because higher risk [9].

While overt hypogammaglobulinemia at CLL diagnosis correlates with disease severity [10]. Ig status in individuals with occult MBL is poorly characterized. Studies lacked information on whether participants harbored MBL at baseline, confounding interpretation of whether Ig alterations precede or accompany early clonal expansion. No studies have examined whether routine clinical Ig measurements, obtained for various indications in real-world practice, can identify individuals likely harboring undiagnosed MBL.

We hypothesized that individuals with prevalent, but undiagnosed clonal expansion or MBL exhibit characteristic Ig profiles detectable through routine clinical testing, even when values remain within established reference ranges. Specifically, we aimed to determine whether low-normal Ig levels in patients undergoing clinical Ig testing identify those likely harboring occult MBL, as evidenced by subsequent CLL diagnosis within 10 years. Understanding these patterns may enable targeted flow cytometry screening in high-yield populations and elucidate immune alterations accompanying early clonal B-cell expansion.

### Study design

The study was based on the STROBE guidelines. This retrospective quantitative observational study aimed to evaluate the association between Ig levels and the development of CLL. A quantitative study design was employed to enable robust statistical assessment of time-to-event outcomes through survival analysis methods. This approach is particularly suited for the evaluation of real-world data routinely generated in clinical practice, as it facilitates the investigation of relationships between clinical biomarkers and patient outcomes while accounting for potential confounding factors. We first checked Ig as continuous value in the Cox regression to avoid bias of categorization. Continuous variables can be categorized considering clinical threshold justified doing so [11, 12] (more information in Supplement 1).

Kaplan-Meier curves and Cox regression were used complementarily: Kaplan-Meier analysis provides a non-parametric visual assessment of survival differences, while Cox regression quantifies the independent effect of Ig levels, adjusting for confounders.

### Patient population

De-identified patient-level data were retrieved from Clalit Health Services (CHS), a large health maintenance organization providing mandatory healthcare coverage to a diverse population. Data were extracted from the CHS central data warehouse, and statistical analyses were performed using Wiser, version alpha [13–15].

The study population included adult patients aged 40–80 years with a diagnosis of CLL between January 1, 2004, and December 31, 2024. Patients who left CHS or were born in another country and later became residents were excluded to ensure complete follow-up. For descriptive analyses comparing baseline characteristics, the ‘Control’ group comprised of randomly age (40–80 years) and sex matched patients (155,700 patients) without diagnosis of CLL. Ig levels (IgG, IgA, IgM), age, and gender were extracted on August 26, 2025.

Patients entered the group based on the first blood sample with Ig levels obtained between age 40 and 80 years. For Cox regression and Kaplan-Meier curve, a sample of patients with at least one IgG with IgA and IgM measurement was selected. Each Ig was dichotomized at the median value of the Ig observed in the CLL cohort 10 years prior to diagnosis. Based on the median Ig values observed in the CLL cohort, we defined two categories for each biomarker: a low-normal group (values below the CLL-specific median) and a higher level group (values at or above the median). All available patients were included given the large real-world dataset, without a predefined sample size calculation. Long-term outcomes and age-stratified risks were assessed.

### CLL definition

The CLL cohort was defined using the ICD-9 diagnostic code 204.1 for CLL. For patients with a prior diagnosis recorded under a broader leukemia category, the onset of CLL was assigned to the earliest documented leukemia-related diagnosis to ensure accurate temporal classification of disease onset.

### Diagnostic validation and laboratory standardization

CLL diagnoses were validated by confirming elevated lymphocyte counts at diagnosis and reduced survival compared to controls taken randomly in the Clalit population, matched for age and sex. Igs were measured using standardized laboratory methods: nephelometry (2007–2018, BN II system, Siemens) and turbidimetry (2018–2024, ARCHITECT, Abbott). Sensitivity analyses confirm consistent results across both periods (Supplement 1).

### Statistical analysis

Time-to-event analyses were conducted from the index date up to 10 years of follow-up. Kaplan-Meier survival analysis was performed to estimate survival probabilities over time. Separate curves were generated for males and females, with 95% confidence intervals plotted. The log-rank test was used to compare survival distributions between groups, and group characteristics were compared using chi-square tests. Statistical significance was defined as *p* < 0.05. Multivariate Cox proportional hazards regression models were used to estimate the independent association between Ig levels and time to CLL development. Age and gender were included as covariates. Patients who were lost to follow-up or did not develop CLL within 10 years were censored. Adjusted hazard ratios (HR) with 95% confidence intervals (CI) were reported. Based on the literature [16]. Ig values were assessed at 10 years prior to the index date, and each Ig was dichotomized at the median value of the Ig observed in the CLL cohort 10 years prior the diagnosis.

To assess robustness to confounding, we performed sensitivity analyses adjusting for clinical conditions associated with altered Ig levels: immunosuppressive medications (systemic corticosteroids, conventional immunosuppressants, biologic therapies), chronic infections (HIV, viral hepatitis, chronic bacterial infections), autoimmune diseases, transplant status, and rituximab exposure (Supplement Methods). To address potential competing risk bias from non-CLL mortality, we performed sensitivity analysis restricted to patients aged 40–70 years, where competing mortality is substantially lower than in the full cohort (ages 40–80 years) (Supplement 2).

### Ethical considerations and data handling

The study adhered to recognized ethical guidelines and was approved by the relevant institutional review board. To protect patient privacy, all data were anonymized before analysis, and no personal identifiers were accessible to investigators.

## Results

The cohort include 294,712 patients and consisted predominantly of individuals aged 60–70 years (28.4%) and 70–80 years (27.4%), with smaller representation in the 40–50 and 50–60 age groups. Males accounted for around 43.27% of the population. Based on the median of the CLL group, a substantial proportion of participants exhibited low-normal Ig levels, most notably lower IgM ( < 70.5 mg/dL) (40.7%) and lower IgG ( < 1120 mg/dL) (41.1%), while lower IgA ( < 187 mg/dL) was present in 30.2%. There were 1571 events (patients diagnosed with CLL) during the study period. Additional information is found in Supplement 1.

### Characteristics of the population

Patients who later developed CLL had markedly lower median and mean IgM, IgG, and IgA levels compared with controls (all *p* < 0.01). They also displayed higher absolute and relative lymphocyte counts at baseline, with both parameters significantly elevated in future CLL cases (*p* < 0.01). Overall, the Ig profile and lymphocyte indices showed clear, statistically significant differences between cases and controls (Table 1).

**Table 1:** A Characteristics of the Population at index date. B: Laboratory of CLL and control data 10 years before diagnosis.

A		
Feature	Category	Number
**(%)**		
Age (years)	40–50	62,059 (21.1)
	50–60	67,362 (22.8)
	60–70	84,022 (28.5)
	70–80	80,746 (27.4)
Sex	Male	127,525 (43.27)
Immunoglobulins	Low IgM ( < 70.5 mg/dL)	125,130 (40.7)
	Low IgA ( < 187 mg/dL)	92,868 (30.2)
	Low IgG ( < 1120 mg/dL)	126,266 (41.1)

B					
Feature	CLL median	Control median	CLL mean (SD)	Control mean (SD)	*p* value*
**IgM**	70.50	98.50	166.81 (291.18)	122.59 (74.48)	<0.01
**IgG**	1120.00	1210.00	1136.18 (442.77)	1279.94 (437.07)	<0.01
**IgA**	187.50	266.00	216.14 (128.66)	268.02 (115.60)	<0.01
**Lym (abs)**	2.40	2.10	3.08 (3.58)	2.20 (0.72)	<0.01
**Lym (%)**	33.10	31.00	34.31 (11.31)	31.06 (8.44)	<0.01

*Kolmogorov–Smirnov (KS) test.

*CLL* Chronic Lymphocytic Leukemia, *Lym* lymphocyte, *abs* absolute.

### Risk of CLL considering level of immunoglobulins

**Fig. 1:**
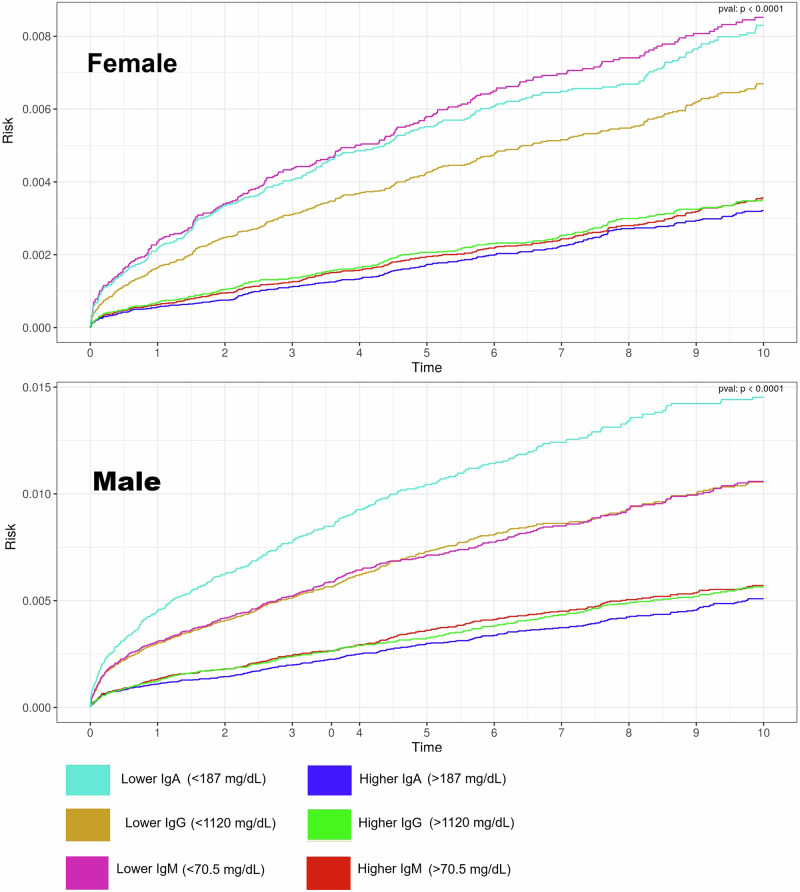
Kaplan-Meier curves: comparison between the lower and the higher level of Ig. Risk of CLL diagnosis, in female and male, by level of IgG, IgM, IgA.

The Kaplan-Meier curves demonstrate clear and consistent differences in cumulative 10-year risk of developing CLL according to baseline Ig levels, stratified by sex. In both females and males, individuals with lower IgA ( < 187 mg/dL) exhibit the highest cumulative incidence over time, with visibly steeper early slopes and progressive separation from all other groups. Lower IgG ( < 1120 mg/dL) and lower IgM ( < 70.5 mg/dL) show intermediate increases in long-term risk, whereas participants with higher Ig levels maintain the lowest and most sπ risk throughout follow-up. Sex-specific patterns reveal that curve separation is most pronounced in males for low IgA and low IgG, while low IgM shows comparable or slightly greater separation in females. All log-rank tests confirmed highly significant differences across Ig groups (*p* < 0.0001) (Fig. 1).

In multivariable Cox regression, increasing age (HR 1.05, CI 1.04–1.05) and male sex (HR 1.55, CI 1.38–1.75) were independently associated with increased 10-year CLL risk. All low-normal Ig levels were significant predictors: low IgM ( < 70.5 mg/dL, HR 1.77, CI 1.57–1.99), low IgG ( < 1120 mg/dL, HR 1.43, CI 1.26–1.62), and low IgA ( < 187 mg/dL, HR 2.62, CI 2.33–2.96) (Table 2).

**Table 2:** Cox regression of immunoglobulins.

Feature	Hazard ratio (95% CI) 0–10 years	Hazard ratio (95% CI) 2–10 years
Age (years)	1.05 (1.04–1.05)	1.04 (1.03–1.05)
Male sex	1.55 (1.38–1.75)	1.44 (1.23–1.69)
Low IgM (<70.5 mg/dL)	1.77 (1.57–1.99)	1.48 (1.26–1.74)
Low IgG (<1120 mg/dL)	1.43 (1.26–1.62)	1.35 (1.14–1.59)
Low IgA (<187 mg/dL)	2.62 (2.33–2.96)	2.02 (1.72–2.37)

To address potential reverse causation, analyses were repeated excluding diagnoses within two years of measurement (2–10 year window). All associations remained statistically significant: low IgA (HR 2.02, CI 1.72–2.37), low IgM (HR 1.48, CI 1.26–1.74), and low IgG (HR 1.35, CI 1.14–1.59).

To evaluate whether Ig-CLL associations depend on baseline lymphocyte counts, we stratified analyses by lymphocyte levels measured concurrently with Ig testing. Patients with low-normal lymphocyte counts ( < 2.2 × 10⁹/L, *n* = 138,730, 177 CLL events, 0.13%) are unlikely to harbor prevalent MBL, while those with high-normal counts (3.0–4.8 × 10⁹/L, *n* = 18,995, 152 CLL events, 0.8%) represent a population enriched for undiagnosed MBL.

Among individuals with low-normal lymphocytes (< 2.2 × 10⁹/L), low IgA remained significantly associated with CLL risk (0–10 years: HR 1.76, CI 1.26–2.47; 2–10 years: HR 1.49, CI 1.00–2.24), whereas low IgM (HR 0.89, CI 0.63–1.27) and low IgG (HR 0.90, CI 0.64–1.26) showed no significant associations (Table 3). In contrast, among patients with high-normal lymphocytes (3.0–4.8 × 10⁹/L), all three Igs showed significant associations: low IgA (HR 2.51, CI 1.76–3.58), low IgM (HR 1.78, CI 1.25–2.52), and low IgG (HR 1.50, CI 1.04–2.17) in the 0–10 years window (Supplement 1).

**Table 3:** Cox regression considering lymphocyte less than 2.2 (×10⁹/L) and lymphocyte between 3 and 4.8 (×10⁹/L).

	lymphocyte less than 2.2 (×10⁹/L)		lymphocyte between 3 and 4.8 (×10⁹/L)	
Feature	0–10 years HR (95% CI)	2–10 years HR (95% CI)	0–10 years HR (95% CI)	2–10 years HR (95% CI)
**Age**	**1.04 (1.02–1.06)**	**1.03 (1.02–1.05)**	**1.05 (1.04–1.07)**	**1.04 (1.02–1.06)**
**Male gender**	**1.44 (1.04–2.00)**	1.32 (0.90–1.95)	**1.95 (1.38–2.75)**	**1.83 (1.15–2.90)**
**IgM** < **70.5 mg/dL**	0.89 (0.63–1.27)	0.83 (0.54–1.26)	**1.78 (1.25–2.52)**	1.51 (0.95–2.41)
**IgG** < **1120 mg/dL**	0.90 (0.64–1.26)	1.00 (0.67–1.49)	**1.50 (1.04–2.17)**	1.55 (0.95–2.53)
**IgA** < **187 mg/dL**	**1.76 (1.26–2.47)**	**1.49 (1.01–2.24)**	**2.51 (1.76–3.58)**	**1.85 (1.15–2.99)**

To evaluate whether associations remain valid using externally defined, clinically relevant thresholds rather than cohort-specific values, we repeated analyses using the lower quartile of standard laboratory reference ranges (IgA <200 mg/dL, IgM <75 mg/dL, IgG <1150 mg/dL).

Using reference range lower quartiles as thresholds, associations remained robust with effect sizes nearly identical to cohort-specific analyses. This remarkable consistency across three independent threshold definitions provides compelling evidence for threshold-independent, dose-dependent biological relationships rather than artifacts of threshold selection. From a clinical perspective, reference range-based thresholds (IgA <200 mg/dL, IgM <75 mg/dL, IgG <1150 mg/dL) offer practical implementation advantages: they are easily identifiable in routine laboratory reports, and represent the lower quartile of normal distributions suitable for selective screening strategies (Table 4, Supplement 1).

**Table 4:** Cox regression based on the lower quartile of normal reference ranges.

Feature	0–10 years HR (95% CI)	2–10 years HR (95% CI)
Age	1.04 (1.04–1.05)	1.04 (1.04–1.05)
Gender	1.56 (1.39–1.75)	1.44 (1.23–1.69)
IgM < 75	1.72 (1.53–1.94)	1.48 (1.26–1.74)
IgG < 1150	1.49 (1.31–1.69)	1.38 (1.17–1.64)
IgA < 200	2.54 (2.25–2.86)	2.06 (1.75–2.42)

### Dose-response relationships between immunoglobulin levels and CLL risk

To assess whether Ig-CLL associations operate across continuous gradients or reflect threshold effects, we performed categorical dose-response analyses using the highest Ig categories as reference groups (IgA >266 mg/dL, IgM >150 mg/dL, IgG >1400 mg/dL) (Table in Supplement 1). All three Ig classes demonstrated steep, monotonic dose-response relationships across the entire clinically normal range.

For IgA, HR increased progressively from 1.63 (95% CI 1.39–1.91) in the 220–266 mg/dL category to 9.25 (95% CI 8.06–10.61) in the <100 mg/dL category, representing a ninefold risk gradient entirely within reference ranges (Supplement 1). IgM showed a fourfold gradient from highest to lowest categories (HR 4.10, 95% CI 3.52–4.76 for <40 mg/dL), while IgG demonstrated a fivefold gradient (HR 5.08, 95% CI 4.27–6.04 for <700 mg/dL) (Supplement 1). Notably, even mid-range values conventionally considered “normal” conferred elevated risk: IgA 150–187 mg/dL (median of CLL cohort) showed HR 2.82 (95% CI 2.44–3.26), while IgA 220–266 mg/dL (median of control cohort) remained significantly elevated at HR 1.63 (95% CI 1.39–1.91) (Supplement 1).

When modeled as continuous variables, each 100 mg/dL decrease in Ig level was associated with increased CLL risk: IgA HR 1.87 (95% CI 1.76–1.99), IgM HR 1.16 (95% CI 1.10–1.23), and IgG HR 1.07 (95% CI 1.06–1.09) (Supplement 1). The continuous associations complement the categorical analyses, confirming genuine dose-dependent biological gradients rather than artifacts of threshold selection.

These findings demonstrate that CLL risk concentrates disproportionately at lower Ig values, with the steepest gradients below cohort medians.

Ig measurements were performed using nephelometry (2007–2018, BN II system, Siemens) or turbidimetry (2018–2024, ARCHITECT system, Abbott), both standardized against international reference preparations. Platform-stratified analyses showed consistent associations across measurement eras (Supplement 2), confirming robustness to methodological variation. Ig-CLL associations remained unchanged after comprehensive covariate adjustment (Supplement 2). HR showed <1% attenuation: IgA 2.61 (95% CI 2.32–2.94) vs 2.62 in primary models; IgM 1.76 (1.57–1.99) vs 1.77; IgG 1.43 (1.26–1.61) vs 1.43. All associations retained statistical significance. Notably, conditions that suppress Ig levels through distinct mechanisms—autoimmune disease (HR 0.77, 95% CI 0.50–1.19), immunosuppressed states (HR 0.85, 95% CI 0.59–1.24), chronic infections (HR 1.13, 95% CI 0.54–2.37), and non-biologic immunosuppressive drugs (HR 1.00, 95% CI 0.52–1.95)—showed null or inverse associations with CLL, arguing strongly against confounding by these conditions. Sensitivity analysis restricted to ages 40–60 and 40–70 years, where competing mortality risk is substantially reduced, showed consistent or stronger Ig-CLL associations (Supplement 2).

## Discussion

### Primary findings and dual-mechanism model

This population-based study demonstrates that low-normal serum Ig levels, particularly IgA, identify individuals who will be diagnosis with CLL up to a decade later. Our lymphocyte-stratified analyses reveal a critical mechanistic distinction: IgM and IgG reductions function as lymphocyte-dependent biomarkers of prevalent undiagnosed MBL, whereas IgA reductions capture both tumor burden and constitutional susceptibility that operates independently of clonal expansion. Among patients with high-normal lymphocyte counts (3.0–4.8 × 10⁹/L), a population enriched for undiagnosed MBL, all three Ig classes showed robust associations (IgM HR 1.78, CI 1.25–2.52; IgG HR 1.50, CI 1.04–2.17; IgA HR 2.51, CI 1.76–3.58). In contrast, among individuals with low-normal lymphocyte counts ( < 2.2 × 10⁹/L) where substantial MBL is unlikely, low IgM and low IgG demonstrated no predictive value (HR 0.89 and 0.90, respectively), while low IgA maintained robust associations (HR 1.76, CI 1.26–2.47). This lymphocyte-dependent pattern for IgM and IgG establishes them as markers of tumor burden, with associations emerging only when lymphocyte elevation suggests substantial clonal populations. Conversely, IgA’s persistent predictive value in the absence of lymphocyte elevation indicates it reflects intrinsic biological characteristics that predispose to lymphoproliferative disorders rather than solely marking existing clonal burden. This dual-mechanism framework advances understanding of preclinical CLL biology and may provide a foundation for integrated risk stratification strategies. We formally tested the dual-mechanism hypothesis through 2×2 stratification by lymphocyte count and Ig level (Supplement 2). IgA demonstrated near-multiplicative interaction (observed HR 2.51 vs predicted 1.85 × 1.24 = 2.29), with persistent associations in normal-lymphocyte patients (HR 1.85, 95% CI 1.45–2.37) supporting constitutional susceptibility independent of clonal burden. In contrast, IgM and IgG associations were lymphocyte-dependent (HRs ≈1.1 in normal-lymphocyte patients), consistent with detection of tumor burden from prevalent MBL.

### IgM and IgG as lymphocyte-dependent markers of tumor burden

Multiple lines of evidence demonstrate that low-normal IgM and IgG levels primarily reflect detection of prevalent but undiagnosed MBL rather than prediction of truly future disease in healthy individuals. First, the future CLL cohort demonstrated substantially elevated baseline lymphocyte counts (mean 3.08 vs 2.20  × 10⁹/L), strongly suggesting that many individuals harbored clonal B-cell populations at the time of Ig measurement. Second, the complete loss of IgM and IgG associations in patients with low-normal lymphocyte counts ( < 2.2 × 10⁹/L) provides evidence that these Igs function as tumor burden markers rather than independent predictors of future malignancy. Third, associations generally strengthened proximal to diagnosis, consistent with progressive immune suppression as clonal burden increases toward clinical thresholds [16].

The biological mechanisms underlying IgM and IgG suppression in MBL and early CLL are established. Hypogammaglobulinemia in advanced CLL results from defective Ig synthesis due to aberrant B-cell receptor signaling, widespread immune dysregulation, and immunosuppressive cytokines within the tumor microenvironment [17]. These mechanisms likely operate at lower intensity during MBL stages, producing the subtle reductions observed in our study. Clonal CD5 + CLL cells suppress Ig secretion and induce apoptosis of healthy plasma cells [18]. with cumulative effects becoming detectable even when clonal populations remain below clinical diagnostic thresholds. The temporal gradient observed in piecewise Cox regression, where IgM and IgG associations were strongest in the 0–4 years window (HR 2.03 and 1.51, respectively) and attenuated at earlier time points, supports this progressive suppression model (Supplement 2). By 10–13 years before diagnosis, neither IgM nor IgG showed robust statistical significance, indicating that their predictive value emerges primarily as MBL clones expand over time.

When adjusted for lymphocyte count as a continuous covariate, IgM and IgG associations showed minimal attenuation (IgM HR 1.63 vs unadjusted 1.77; IgG HR 1.44 vs unadjusted 1.43), confirming they provide independent prognostic information beyond simple lymphocyte elevation (Supplement 2). This suggests that Ig levels capture functional immune suppression and aspects of clonal biology not fully reflected in total lymphocyte number. The combination of elevated lymphocytes and suppressed Igs likely represents a higher-burden MBL phenotype with greater malignant potential.

### IgA as a marker of constitutional susceptibility and tumor burden

In contrast to IgM and IgG, low IgA demonstrates a distinct biological profile indicating it captures constitutional susceptibility independent of clonal burden. The critical evidence comes from lymphocyte-stratified analyses: even in patients with low-normal lymphocyte counts ( < 2.2 × 10⁹/L) where substantial MBL is unlikely, low IgA ( < 187 mg/dL) maintained robust associations with subsequent CLL development (HR 1.76, CI 1.26–2.47). This persistent predictive value in the absence of lymphocyte elevation indicates that low IgA reflects intrinsic biological characteristics that predispose to lymphoproliferative disorders rather than solely marking existing clonal burden.

The constitutional nature of this association is further supported by temporal dynamics revealed through piecewise Cox regression. While all Igs showed stronger associations proximal to diagnosis (IgA HR 3.18 at 0–4 years), IgA uniquely maintained associations extending 10–13 years prior to clinical CLL (HR 1.73, CI 1.11–2.71) (Supplement 2). Nevertheless, this extended predictive horizon, particularly when contrasted with the complete loss of IgM/IgG associations at this interval, argues against pure detection of preclinical disease and instead supports identification of stable genetic or constitutional traits that confer long-term risk.

The biological plausibility of IgA-specific constitutional susceptibility is supported by the genetics of selective IgA deficiency. Selective IgA deficiency demonstrates strong genetic predisposition with susceptibility loci in the MHC region, specifically HLA-B8, DR3, and DQ2 haplotypes [19, 20]. Critically, individuals with IgA deficiency exhibit increased risk of lymphoma and leukemia [21]. suggesting shared genetic architecture between IgA production capacity and lymphoproliferative disorder susceptibility. Our findings extend this relationship by demonstrating that even low-normal IgA levels, representing form fruste of IgA deficiency due to partial penetrance of susceptibility alleles, identify individuals at elevated CLL risk decades before diagnosis.

Importantly, IgA’s dual mechanism is evident in its behavior across lymphocyte strata. While maintaining significant associations in low-lymphocyte individuals (HR 1.76), the effect size increased substantially in high-lymphocyte individuals (HR 2.51), indicating that IgA captures both constitutional predisposition and acquired tumor-related suppression. This combination explains why IgA demonstrates both long-term predictive value in phenotypically healthy individuals and intensified associations in MBL-enriched populations. When adjusted for lymphocyte count as a continuous covariate, IgA associations showed minimal attenuation (adjusted HR 2.42 vs unadjusted HR 2.62), confirming independent prognostic value beyond lymphocyte elevation and supporting its role as a marker of constitutional immune capacity. Longitudinal Ig trajectories provide visual evidence for IgA’s constitutional role: IgA shows early divergence from controls beginning 12-15 years before diagnosis with consistent linear decline (R² = 0.728), whereas IgG remains stable until 5 years before diagnosis with late-stage acceleration (R² = 0.547) (supplement 1). This early IgA divergence at the most distant measurable time points supports baseline constitutional characteristics rather than solely acquired suppression from clonal burden. Nevertheless, the extended temporal reach of IgA associations (detectable 10–13 years before diagnosis) does not definitively prove lifelong constitutional susceptibility versus very early clonal expansion, as we lack Ig measurements from early adulthood.

### Validation through dose-response relationships

The genuine biological nature of Ig-CLL relationships is strongly supported by continuous dose-response gradients across the entire normal range (Supplement 2). Categorizing Ig levels into three groups (below CLL median, between medians, above control median) revealed striking monotonic relationships. For IgA, compared to low-normal values (<187 mg/dL), intermediate values (187–266 mg/dL) conferred 50% risk reduction (HR 0.50, CI 0.43–0.57), while high-normal values (>266 mg/dL) provided 71% risk reduction (HR 0.29, CI 0.25–0.34), representing an approximately 3.4-fold risk gradient entirely within clinically defined normal ranges. IgM and IgG demonstrated similar but less pronounced gradients (1.8-fold and 1.4-fold respectively). This non-linear distribution demonstrates that absolute CLL risk concentrates disproportionately in the lowest quartile, with the steepest gradient occurring below the CLL cohort median. The intermediate group represents a transition zone with attenuated but clinically meaningful risk elevation, while high-normal values approach baseline population risk. Notably, 30.2% of our cohort exhibited low-normal IgA ( < 187 mg/dL) at baseline, remarkably similar to the 30% prevalence of IgA deficiency reported at CLL diagnosis [5]. suggesting that low-normal IgA may represent an early manifestation along a continuum from constitutional susceptibility to overt immunodeficiency as clonal burden increases. Notably, 88.9% of patients who developed CLL exhibited at least one low-normal Ig during the preceding decade (supplement 1).

The robustness of associations across multiple analytical approaches further validates biological authenticity. Findings remained consistent using three independent threshold definitions: CLL cohort medians (primary analysis, IgA HR 2.62), using control cohort medians (IgA <266 mg/dL, HR 2.63), and reference range lower quartiles (IgA <200 mg/dL, HR 2.54). This remarkable consistency across threshold definitions provides compelling evidence for threshold-independent, dose-dependent biological relationships rather than artifacts of threshold selection. The exclusion of patients with overt Ig deficiencies (IgA <70 mg/dL, IgM <40 mg/dL, IgG <700 mg/dL) preserved significant associations (IgA HR 2.17), demonstrating that findings derive from subtle variations within normal ranges rather than pathological immunodeficiency. Sensitivity analyses censoring for unrelated diseases (hyperparathyroidism and schizophrenia) showed no association with Ig levels (all HRs ≈1.0), confirming specificity for hematologic outcomes (Supplement 2).

### Convergence with extended preclinical CLL natural history

These findings converge with emerging evidence that CLL develops through extended preclinical phases. High-risk CLL subtypes identified by B-cell receptor characteristics are detectable up to 16 years prior to diagnosis [22]. and CLL-associated genetic mutations can be present decades before clinical disease [23]. Our demonstration that Ig alterations identify at-risk individuals more than a decade before diagnosis reinforces the concept of CLL as a disease with subtle early immune dysregulation, likely originating with MBL or pre-MBL states. The prevalence of low-normal Igs in our CLL cohort is striking: of 1571 patients who developed CLL within 10 years, 1397 (88.9%) had at least one Ig class in the low-normal range during the decade preceding diagnosis. This high proportion demonstrates that Ig alterations are nearly ubiquitous in preclinical CLL. MBL is present in approximately 6-13% of individuals over age 40 and progresses to clinical CLL at a rate of 1–2% per year [6, 7]. Recent evidence demonstrates that MBL may be present decades before CLL diagnosis [8].

### Clinical implications and future directions

The continuous dose-response relationships observed across all Ig classes challenge traditional binary normal/abnormal classifications in clinical practice. The ninefold risk gradient for IgA from highest to lowest normal categories indicates that individuals with low-normal values (e.g., 150–200 mg/dL) harbor substantially elevated CLL risk despite values well above clinical deficiency thresholds. This suggests that routine Ig measurements, obtained for various clinical indications, capture quantitative variation in immune function that reflects both genetic predisposition and early clonal expansion.

However, the modest absolute risk even in high-risk groups (10-year cumulative incidence 1.45% in males with IgA <100 mg/dL vs 0.24% in controls) indicates that widespread systematic screening based solely on Ig patterns would be premature. Rather, these findings suggest Ig levels should be integrated into multivariable risk models combining lymphocyte counts, family history, and other established risk factors to identify individuals who might benefit from targeted monitoring or enrollment in early detection studies.

### Limitations

Several limitations warrant consideration. First, while we employed standard Cox regression with censoring at death, formal Fine-Gray competing risk models accounting for the subdistribution hazard were not available in our analytical platform. However, sensitivity analysis restricted to ages 40–70 and 40–60 years—where competing mortality is substantially lower—showed consistent or stronger associations, arguing against competing risks as an alternative explanation. Second, CLL identification relied on ICD-9 coding rather than gold-standard flow cytometry confirmation, introducing potential diagnostic misclassification. Nevertheless, validation analyses confirmed elevated lymphocyte counts at diagnosis (mean 12.29 vs 2.17 × 10⁹/L in controls) and reduced survival in the CLL cohort, supporting diagnostic validity. Third, Ig measurements were clinically indicated rather than obtained through systematic population screening, potentially enriching our cohort for individuals with subclinical hematologic or inflammatory conditions. However, the high proportion of apparently healthy individuals (mean baseline lymphocyte 2.20 × 10⁹/L in controls) and preserved associations after excluding overt immunodeficiency suggest findings apply to routine clinical populations. Fourth, lack of baseline flow cytometry prevents definitive determination of MBL prevalence, limiting our ability to distinguish whether low Igs precede or coincide with clonal expansion. However, lymphocyte-stratified analyses provide strong indirect evidence, with IgA associations persisting even in normal-lymphocyte patients where substantial MBL is unlikely. Fifth, findings may not generalize to populations with different genetic backgrounds, healthcare access patterns, or laboratory practices. Finally, despite comprehensive covariate adjustment, residual confounding by unmeasured factors (e.g., environmental exposures) cannot be fully excluded, though negative control analyses showing null associations with unrelated outcomes (hyperparathyroidism, schizophrenia) provide reassurance against major unmeasured confounding. Despite these limitations, the large sample size, extended follow-up, consistency across multiple sensitivity analyses, and concordance with established CLL biology provide confidence in the fundamental conclusions.

## Conclusions

This population-based study demonstrates that Ig levels across the entire clinically normal range show continuous, dose-dependent associations with CLL diagnosis up to a decade later, with 3–4 fold risk gradients between low-normal and high-normal values. Critically, lymphocyte-stratified analyses reveal these associations operate through a dual mechanism: IgM and IgG reductions function as lymphocyte-dependent markers that detect prevalent undiagnosed MBL (tumor burden), whereas IgA reductions capture both tumor burden and constitutional susceptibility identifiable even before substantial clonal expansion. The findings challenge conventional views that Ig abnormalities are primarily late-stage phenomena, revealing instead that subtle immune dysregulation within clinically normal ranges signals underlying hematologic abnormalities years before diagnosis. While these insights suggest potential for targeted screening strategies integrating Ig profiling with lymphocyte counts to identify high-risk individuals harboring undiagnosed MBL, prospective validation demonstrating clinical benefit from early detection is required before widespread implementation.

## Supplementary information

supplement 2

supplement 1

## Data Availability

The data supporting this study are available through the WISER platform or from the corresponding author upon reasonable request. All WISER-derived data are provided in aggregated form only and not at the individual-patient level, in accordance with ethical and privacy requirements, as detailed in the manuscript.
