# Supplementary material for: Low-normal immunoglobulin suggests monoclonal B-cell lymphocytosis and constitutional CLL susceptibility: a dual-mechanism analysis
Source: Leukemia. 2026 Jun 10;40(8):1668–75. doi: 10.1038/s41375-026-02987-2 (PMC13421330; doi:10.1038/s41375-026-02987-2)
Supplement: Supplementary file 1 — supplement 2 [file 41375_2026_2987_MOESM1_ESM.docx]

**Supplement (2)**

**Sub-analysis**

Piecewise Cox proportional hazards ratio

To evaluate the temporal stability of associations and assess whether immunoglobulin (Ig)-CLL relationships reflect stable constitutional traits versus progressive immune dysregulation, we performed piecewise Cox proportional hazards analysis across five-time intervals. This approach tests the proportional hazards assumption and reveals whether associations strengthen, attenuate, or remain constant as clinical diagnosis approaches.

| **Feature** | **0–4 years**  **HR (95% CI)** | **4–7 years**  **HR (95% CI)** | **7–10 years**  **HR (95% CI)** | **10 - 13years**  **HR (95% CI)** | **13 - 15 years**  **HR (95% CI)** |
| --- | --- | --- | --- | --- | --- |
| **Age** | **1.04 (1.03–1.05)** | **1.05 (1.03–1.06)** | **1.01 (1.00–1.03)** | **1.03 (1.01–1.05)** | 1.00 (0.97–1.03) |
| **Male sex** | **1.64 (1.42–1.89)** | **1.40 (1.08–1.83)** | 1.13 (0.82–1.56) | **1.69 (1.09–2.62)** | **2.28 (1.16–4.46)** |
| **Low IgM (<70.5 mg/dL)** | **2.03 (1.75–2.35)** | **1.33 (1.02–1.74)** | 1.32 (0.95–1.83) | 1.09 (0.69–1.70) | 0.72 (0.34–1.49) |
| **Low IgG (<1120 mg/dL)** | **1.51 (1.29–1.76)** | **1.36 (1.04–1.80)** | 1.22 (0.88–1.70) | **1.77 (1.11–2.81)** | 1.53 (0.77–3.04) |
| **Low IgA (<187 mg/dL)** | **3.18 (2.74–3.69)** | **1.97 (1.51–2.58)** | **1.64 (1.18–2.27)** | **1.73 (1.11–2.71)** | 1.34 (0.66–2.72) |

The piecewise analysis reveals distinct temporal patterns across Ig classes, providing mechanistic insights into the relationship between immune dysregulation and CLL development. Low IgA demonstrates the most robust and persistent associations, with hazard ratios of 3.18 (2.74-3.69) at 0-4 years, declining progressively to 1.97 (1.51-2.58) at 4-7 years, 1.64 (1.18-2.27) at 7-10 years, and 1.73 (1.11-2.71) at 10-13 years, before losing statistical significance beyond 13 years; this likely reflecting reduced statistical power rather than true null effect given the maintained point estimate of 1.34. This temporal gradient supports a model of progressive immune suppression as clonal burden increases over time, with the strongest associations appearing immediately before clinical diagnosis when MBL is most likely transitioning to overt CLL. Notably, however, the persistence of statistically significant associations extending 10-13 years before diagnosis indicates that a substantial subset of patients harbor either high-burden MBL for extended periods or possess constitutional immune characteristics predisposing to early disease. Low IgM and IgG show similar but less pronounced temporal patterns, with associations strongest in the 0-4 year window and generally attenuating at earlier time points. The loss of significance for all Ig classes beyond 13 years likely reflects the combination of reduced sample size with available measurements at such distant time points and the biological reality that Ig alterations become increasingly subtle at greater temporal distances from clinical diagnosis. These temporal dynamics support our interpretation that low-normal Ig levels primarily detect prevalent but undiagnosed MBL rather than independently predicting future disease in truly healthy individuals, while also suggesting a secondary component of genetic predisposition among those with very low values detected many years before diagnosis.

Cox regression for 10 years (per laboratory)

| **Feature** | **HR (95% CI)**  **0 - 10 years** | **HR (95% CI)**  **2 – 10 years** |
| --- | --- | --- |
| **Age (years)** | **1.05 (1.04–1.05)** | **1.04 (1.04–1.05)** |
| **IgM < 70 mg/dL** | **1.13 (1.02–1.25)** | 0.92 (0.81–1.04) |
| **IgG < 1120 mg/dL** | 0.94 (0.85–1.03) | 1.02 (0.89–1.16) |
| **IgA < 187 mg/dL** | **1.72 (1.55–1.90)** | **1.47 (1.34–1.61)** |
| **Male sex** | **1.54 (1.43–1.65)** | **1.47 (1.28–1.69)** |

Relative risk at 10 years compared to general control (Based on risk of the Kaplan-Meir curve)

| **Feature** | **Male RR (95% CI)** | **Female RR (95% CI)** |
| --- | --- | --- |
| **IgG < 1120 mg/dL** | 4.18 (2.47–10.11) | 3.89 (2.30–9.82) |
| **IgM < 70.5 mg/dL** | 4.19 (2.41–10.17) | 4.95 (2.86–11.89) |
| **IgA < 187 mg/dL** | 5.75 (3.31–13.91) | 4.82 (2.76–11.62) |

**Sub-analysis: gender**

To evaluate whether Ig -CLL associations differ by sex, we performed stratified analyses examining both the relative risk between sexes within low Ig groups and sex-specific hazard ratios. While male sex is an established CLL risk factor (overall HR 1.55 in our cohort), it remains unclear whether this male predominance is uniform across all Ig levels or whether low Ig states confer differential risk by sex. Understanding sex-specific effects has clinical implications for risk stratification and may provide insights into whether hormonal, genetic, or immunological factors modify the relationship between immune dysregulation and CLL development.

Relative Risk of male vs female in the lower immunoglobulin Groups (10-Year Risk)

| **Feature** | **Male vs Female RR (95% CI)** |
| --- | --- |
| **IgG < 1120 mg/dL** | 1.58 (1.36–1.82) |
| **IgM < 70.5 mg/dL** | 1.24 (1.05–1.47) |
| **IgA < 187 mg/dL** | 1.74 (1.49–2.05) |

Cox regression 0 to 10 years (per sex)

Multivariable Cox Proportional Hazards Models for CLL – only patient with the first Ig were included (Patients between 40 and 80 years old, exclusion if quit CHS or born in another country). Male 123,117 patients (832 patients with CLL). Female 171,595 patients (739 patients with CLL).

| **Feature** | **HR (95% CI) - Male** | **HR (95% CI) - Female** |
| --- | --- | --- |
| **Age (years)** | **1.04 (1.03–1.05)** | **1.04 (1.03–1.05)** |
| **IgM < 70 mg/dL** | **1.53 (1.29–1.81)** | **2.06 (1.72–2.47)** |
| **IgG < 1120 mg/dL** | **1.48 (1.24–1.76)** | **1.39 (1.15–1.68)** |
| **IgA < 187 mg/dL** | **2.69 (2.27–3.19)** | **2.49 (2.07–2.99)** |

The gender-stratified analyses reveal complex sex-specific patterns. Males with low-normal Ig demonstrate 1.24-1.74 fold higher relative risk compared to females with similarly low values, with the strongest sex differential observed for low IgA (RR 1.74, CI 1.49-2.05). However, examination of sex-specific hazard ratios reveals an unexpected pattern: low IgM shows substantially stronger associations in females (HR 2.06, CI 1.72-2.47) compared to males (HR 1.53, CI 1.29-1.81), while IgA and IgG demonstrate similar effect sizes across sexes. This apparent paradox, characterized by higher absolute risk in males within low- Ig groups despite stronger IgM hazard ratios in females, likely reflects baseline sex differences in both CLL incidence and Ig distributions. Females have lower baseline CLL risk, so the presence of low IgM may represent a more selective marker of high-risk phenotypes in women, whereas in men, low IgM may be more prevalent and less specific. The consistent strength of IgA associations across both sexes (HR 2.69 in males, 2.49 in females) reinforces its robust predictive value independent of sex-specific factors, supporting IgA as the most reliable Ig marker for CLL risk stratification in both men and women.

**Sub-analysis: Age**

Cox regression 0 to 10 years considering the age

Because serum Ig levels tend to be higher in younger individuals, we conducted an age-stratified analysis to confirm that the associations remained statistically significant across different age groups. Participants were divided into two predefined categories—40–60 years and 60–80 years—and all analyses were repeated within each stratum to evaluate the consistency of the findings across age groups

| **Feature** | **HR (95% CI)**  **40-60 years** | **HR (95% CI)**  **60-80 years** |
| --- | --- | --- |
| **Age** | 1.07 (1.05–1.10) | 1.02 (1.01–1.03) |
| **Gender** | 1.39 (1.09–1.77) | 1.60 (1.40–1.83) |
| **IgM < 70.5 mg/dL** | 1.98 (1.55–2.53) | 1.70 (1.49–1.95) |
| **IgG < 1120 mg/dL** | 1.59 (1.23–2.04) | 1.37 (1.19–1.58) |
| **IgA < 187 mg/dL** | 2.43 (1.90–3.10) | 2.70 (2.36–3.10) |

**Sub-analysis: Alternative Immunoglobulin Thresholds**

Our primary analysis used cohort-specific median values from patients who developed CLL as cut-points (IgA 187 mg/dL, IgM 70.5 mg/dL, IgG 1120 mg/dL). While this data-driven approach maximizes statistical power, it has limitations for clinical application and external validity.

Cox regression based on the Median of the **control group (more than the median) and the values between the 2 medians (median of CLL and median of control)**

To comprehensively evaluate the dose-response relationship between Ig levels and CLL risk across the entire normal range, we performed analysis using both CLL cohort medians (IgA 187 mg/dL, IgM 70.5 mg/dL, IgG 1120 mg/dL) and control cohort medians (IgA 266 mg/dL, IgM 98.5 mg/dL, IgG 1210 mg/dL) as cut-points to create 2 groups.

| **Feature** | **0–10 years HR (95% CI)** | **2–10 years HR (95% CI)** |
| --- | --- | --- |
| **Age** | **1.04 (1.04–1.05)** | **1.04 (1.03–1.05)** |
| **Gender** | **1.56 (1.39–1.75)** | **1.45 (1.24–1.70)** |
| **IgM 70.5–98.5 mg/dL** | **0.58 (0.49–0.68)** | **0.63 (0.51–0.79)** |
| **IgG 1120–1210 mg/dL** | **0.77 (0.64–0.94)** | **0.78 (0.60–1.02)** |
| **IgA 187–266 mg/dL** | **0.50 (0.43–0.57)** | **0.67 (0.56–0.80)** |
| **IgM >98.5 mg/dL** | **0.55 (0.48–0.63)** | **0.69 (0.58–0.82)** |
| **IgG >1210 mg/dL** | **0.72 (0.63–0.83)** | **0.79 (0.66–0.95)** |
| **IgA >266 mg/dL** | **0.29 (0.25–0.34)** | **0.36 (0.30–0.45)** |

Compared to the reference group (values below the CLL cohort median), both intermediate values (between the two medians) and high values (above the control median) demonstrated protective associations, with progressively stronger protection at higher Ig levels. For IgA, intermediate values (187-266 mg/dL) showed HR 0.50 while high values (>266 mg/dL) showed HR 0.29, revealing a steep monotonic gradient entirely within the clinically normal range. This pattern demonstrates continuous dose-response relationships rather than threshold effects.

Cox regression based on the Median of the **control group (less than)**

To assess whether associations remain robust when using clinically higher thresholds that include a broader population segment, we repeated analyses using control cohort medians as cut-points (IgA <266 mg/dL, IgM <98.5 mg/dL, IgG <1210 mg/dL).

| **Feature** | **0–10 years HR (95% CI)** | **2–10 years HR (95% CI)** |
| --- | --- | --- |
| **Age** | **1.04 (1.04–1.05)** | **1.04 (1.03–1.05)** |
| **Male gender** | **1.58 (1.41–1.78)** | **1.48 (1.26–1.73)** |
| **IgM < 98.5 mg/dL** | **1.56 (1.37–1.77)** | **1.27 (1.07–1.49)** |
| **IgG < 1210 mg/dL** | **1.47 (1.29–1.68)** | **1.30 (1.09–1.54)** |
| **IgA < 266 mg/dL** | **2.63 (2.27–3.05)** | **2.33 (1.92–2.83)** |

Using control cohort medians as thresholds, associations remained robust with dose-response relationships across the normal Ig range. Low IgA (<266 mg/dL) showed nearly identical hazard ratios to the primary analysis (HR 2.63 vs 2.62), demonstrating threshold-independent, continuous biological gradients rather than sharp cutoffs. The persistence of associations in the 2-10 year window argues against reverse causation and suggests constitutional variation in Ig production capacity influences CLL susceptibility across the normal spectrum. This continuous risk pattern, validated across multiple threshold definitions, indicates Ig levels should be integrated with other risk factors (lymphocyte counts, family history) in multivariable assessment rather than applied as dichotomous screening thresholds

"Reconciling Binary and Two-Category Analyses"

Readers may note an apparent discrepancy between binary threshold analyses and two-category analyses. In binary analyses, both low thresholds show significant increased risk: IgA <187 mg/dL (CLL median) shows HR 2.62, and IgA <266 mg/dL (control median) shows HR 2.63. However, in the two-category analysis with <187 mg/dL as reference, intermediate values (187-266 mg/dL) show HR 0.50, which appears "protective." These findings are statistically consistent and reflect different reference categorizations, with adequate statistical power across all groups.

*Sample distribution and event rates:*

IgA <187 mg/dL: n=90,771 (30.8%), 920 events (1.01% incidence)

IgA 187-266 mg/dL: n=85,088 (28.9%), 360 events (0.42% incidence)

IgA >266 mg/dL: n=118,260 (40.1%), 289 events (0.24% incidence)

The narrow confidence intervals for all categories (HR 0.50, 95% CI 0.43-0.57 for intermediate; HR 0.29, 95% CI 0.25-0.34 for high) despite substantial sample sizes demonstrate adequate statistical power, confirming these represent genuine biological gradients rather than statistical artifacts.

*Binary analyses (comparing to high-normal reference ≥266 mg/dL):*

IgA <187 mg/dL vs ≥266 mg/dL: HR 2.62 (1.01% vs 0.24% incidence → 4.2-fold difference)

IgA <266 mg/dL vs ≥266 mg/dL: HR 2.63 (combines 1.01% and 0.42% groups vs 0.24%)

*Two-category analysis (comparing to lowest reference <187 mg/dL):*

IgA 187-266 mg/dL vs <187 mg/dL: HR 0.50 (0.42% vs 1.01% incidence)

IgA >266 mg/dL vs <187 mg/dL: HR 0.29 (0.24% vs 1.01% incidence)

**Mathematical reconciliation:** The HR 0.50 for intermediate values does not indicate absolute protection—it reflects a 58% relative risk reduction compared to the highest-risk group (from 1.01% to 0.42% incidence), while still conferring 1.75-fold higher risk than high-normal values (0.42% vs 0.24%). When the binary analysis combines all values <266 mg/dL (representing 59.7% of the cohort with weighted incidence ~0.73%), the resulting HR 2.63 compared to ≥266 mg/dL (0.24% incidence) accurately reflects the 3.0-fold risk difference. The nearly identical HRs for the two binary thresholds (2.62 vs 2.63) occur because the intermediate group, despite having 58% lower risk than the very lowest values, still carries substantially elevated risk compared to high-normal values, contributing meaningfully to the overall below-median risk elevation.

**Critical insight:** Both binary analyses show nearly identical hazard ratios despite different thresholds because even the intermediate group (187-266 mg/dL) maintains clinically significant risk elevation (0.42% vs 0.24% baseline). The apparent "protective" HR 0.50 is an artifact of reference group selection: intermediate values have half the risk of very low values but still 1.75 times the risk of high-normal values. The steep gradient from lowest to intermediate categories (1.01% to 0.42%, a 2.4-fold decrease) combined with the more modest gradient from intermediate to high (0.42% to 0.24%, a 1.75-fold decrease) demonstrates non-linear risk distribution across the normal range.

**Clinical implications:** This non-uniform distribution demonstrates that CLL risk concentrates disproportionately in the lowest IgA quartile (<187 mg/dL, 1.01% incidence), with the steepest risk gradient occurring below this threshold. The intermediate group (187-266 mg/dL, 0.42% incidence) represents a transition zone with attenuated but still clinically meaningful risk elevation (1.75-fold above baseline), while high-normal values (>266 mg/dL, 0.24% incidence) approach baseline population risk. The overall 4.2-fold risk difference between the lowest and highest categories supports continuous biological relationships where Ig production capacity correlates with CLL/MBL susceptibility. Very low values likely reflect higher-burden MBL or more severe constitutional immune dysregulation, intermediate values may indicate lower-burden MBL or milder susceptibility, and high-normal values suggest preserved immune function with minimal clonal burden.

**Sub-analysis: comparison considering the lymphocytes**

Cox regression Ig and lymphocyte between 3 and 4.8 (×10⁹/L)

To evaluate Ig -CLL associations in a population highly enriched for prevalent but undiagnosed MBL, we restricted analysis to patients with high-normal lymphocyte counts (3.0-4.8 × 10⁹/L, n=18,995) measured concurrently with Ig testing.

| **Feature** | **0–10 years HR (95% CI)** | **2–10 years HR (95% CI)** | **Feature** | **0–10 years HR (95% CI)** | **2–10 years HR (95% CI)** |
| --- | --- | --- | --- | --- | --- |
| **Age** | **1.05 (1.04–1.07)** | **1.04 (1.02–1.06)** | **Age** | **1.06 (1.04–1.07)** | **1.04 (1.02–1.07)** |
| **Male gender** | **1.95 (1.38–2.75)** | **1.83 (1.15–2.90)** | **Male gender** | **1.97 (1.40–2.79)** | **1.87 (1.18–2.97)** |
| **IgM < 70.5 mg/dL** | **1.78 (1.25–2.52)** | 1.51 (0.95–2.41) | **IgM < 98.5 mg/dL** | **1.51 (1.03–2.20)** | 1.19 (0.74–1.93) |
| **IgG < 1120 mg/dL** | **1.50 (1.04–2.17)** | 1.55 (0.95–2.53) | **IgG < 1210 mg/dL** | **1.58 (1.07–2.33)** | 1.68 (1.00–2.84) |
| **IgA < 187 mg/dL** | **2.51 (1.76–3.58)** | **1.85 (1.15–2.99)** | **IgA < 266 mg/dL** | **2.02 (1.36–3.01)** | 1.52 (0.92–2.50) |

In patients with high-normal lymphocyte counts (3.0-4.8 × 10⁹/L) likely enriched for MBL, all Ig classes showed robust associations with larger effect sizes than the overall cohort. Critically, comparing thresholds revealed staged Ig suppression: very low IgA (<187 mg/dL) remained significant in the 2-10 year window (HR 1.85, CI 1.15-2.99), while intermediate-low IgA (<266 mg/dL) lost significance (HR 1.52, CI 0.92-2.50). This pattern suggests mild suppression emerges in the 0-2 years preceding diagnosis as clones expand, while severe suppression reflects higher-burden MBL detectable years earlier. These findings support progressive Ig decline correlating with clonal burden and temporal proximity to clinical diagnosis.

Cox regression Ig and lymphocyte between 0.8 and 3.6 (×10⁹/L)

To evaluate Ig -CLL associations in a population with normal lymphocyte (based on the mean+/- SD of lymphocyte 10 years before), we restricted analysis to patients with normal lymphocyte counts (0.8-3.6 × 10⁹/L, n=18,995) measured concurrently with Ig testing.

| **Feature** | **0–10 years HR (95% CI)** | **2–10 years HR (95% CI)** |
| --- | --- | --- |
| Age (per year) | **1.05 (1.03–1.06)** | **1.04 (1.02–1.05)** |
| Male gender | **1.60 (1.26–2.03)** | **1.46 (1.09–1.95)** |
| IgM < 70.5 mg/dL | 1.11 (0.86–1.42) | 1.02 (0.76–1.39) |
| IgG < 1120 mg/dL | 1.10 (0.86–1.42) | 1.11 (0.82–1.49) |
| **IgA < 187 mg/dL** | **1.85 (1.45–2.37)** | **1.50 (1.11–2.03)** |

**Sub-analysis: considering special situations**

Cox regression excludes hypogammaglobulinemia

To ensure that our findings reflect associations within the clinically normal range rather than being driven by frank immunodeficiency, we performed sensitivity analysis excluding patients with overt hypogammaglobulinemia (IgG <700 mg/dL), IgA deficiency (<70 mg/dL), or IgM deficiency (<40 mg/dL) based on standard clinical reference ranges. By restricting to patients with all Ig values above deficiency thresholds (n=262,032, with 1,029 CLL cases), we test whether associations persist when the cohort is limited to true "low-normal" rather than "abnormally low" Ig levels.

| **Feature** | **HR (95% CI) 0-10 years** | **HR (95% CI) 2-10 years** |
| --- | --- | --- |
| **Age** | **1.04 (1.04 – 1.05)** | **1.52 (1.26–1.82)** |
| **Male sex** | **1.64 (1.43 – 1.88)** | **1.04 (1.03–1.04)** |
| **IgM < 70.5 mg/dL** | **1.37 (1.19 – 1.59)** | 1.14 (0.94–1.39) |
| **IgG < 1120 mg/dL** | **1.26 (1.09 – 1.45)** | **1.23 (1.02–1.49)** |
| **IgA < 187 mg/dL** | **2.17 (1.88 – 2.50)** | **1.67 (1.38–2.02)** |

After excluding all patients with overt Ig deficiencies (IgA <70 mg/dL, IgM <40 mg/dL, IgG <700 mg/dL), associations remained statistically significant, confirming findings reflect subtle variations within the normal range rather than frank immunodeficiency. Low IgA (<187 mg/dL) maintained the strongest association (HR 2.17 at 0-10 years), with low IgM (HR 1.37) and low IgG (HR 1.26) also significant, though modestly attenuated compared to the full cohort. This preservation after excluding ~11% of patients (32,680 with overt deficiencies) demonstrates that Ig -CLL relationships operate across a continuum from low-normal to high-normal rather than as a deficient versus normal dichotomy. The modest attenuation suggests frank immunodeficiency contributes to associations (likely reflecting substantial MBL), but the majority of signal derives from low-normal values. Critically, routine measurements revealing low-normal Ig, well above clinical deficiency thresholds, nonetheless identify individuals with elevated CLL/MBL risk. These results support that Ig production capacity exists on a quantitative spectrum related to CLL susceptibility, with genetic and acquired factors influencing individual positioning along this continuum.

Cox regression considering lymphocyte as continuous variable (0 – 10 years)

To evaluate whether lymphocyte count confounds or mediates the relationship between Ig levels and CLL risk, we repeated the primary analysis including absolute lymphocyte count as a continuous covariate in the Cox regression model. This approach tests whether Ig associations are independent of lymphocyte elevation or whether they are explained by the correlation between low Ig and higher lymphocyte counts (both potentially reflecting MBL).

| **Feature** | **Hazard Ratio (95% CI)** |
| --- | --- |
| **Male sex** | **1.47 (1.27–1.71)** |
| **Age** | **1.04 (1.03–1.04)** |
| **IgM < 70.5 mg/dL** | **1.63 (1.40–1.91)** |
| **IgG < 1120 mg/dL** | **1.44 (1.23–1.69)** |
| **IgA < 187 mg/dL** | **2.42 (2.08–2.83)** |
| **Absolute lymphocyte count** | **1.03 (1.02–1.03)** |

Ig associations remain statistically significant and clinically robust after adjusting for lymphocyte count, demonstrating that they provide independent prognostic information beyond lymphocytosis alone. Low IgA retains an adjusted HR of 2.42 (2.08-2.83), low IgM shows HR 1.63 (1.40-1.91), and low IgG shows HR 1.44 (1.23-1.69), all with minimal attenuation compared to models without lymphocyte adjustment. The lymphocyte count itself shows HR 1.03 (1.02-1.03) per 1 × 10⁹/L increase, indicating that each 1-unit increase in lymphocyte count confers approximately 3% increased CLL risk. Remarkably, the Ig associations are substantially stronger than the lymphocyte effect: low IgA confers 142% increased risk (HR 2.42) even after accounting for lymphocyte count, suggesting that IgA levels capture additional biological information: likely reflecting severity of immune dysregulation, genetic predisposition, or aspects of clonal biology not fully reflected in total lymphocyte number. The independence of Ig and lymphocyte associations suggests they represent complementary rather than redundant risk markers. Lymphocyte count primarily reflects clonal burden (more circulating monoclonal cells), while Ig levels reflect functional immune suppression and possibly constitutional immune capacity.

Cox regression censoring for unrelated diseases (0 -10 years)

To ensure specificity, we censored for unrelated disease.

| **Feature** | **Hyperparathyroidism** | **Schizophrenia** |
| --- | --- | --- |
| **Age** | **1.01 (1.01–1.02)** | 0.97 (0.97–0.98) |
| **Gender** | 0.30 (0.26–0.34) | **1.26 (1.08–1.46)** |
| **IgM < 70.5 mg/dL** | 0.94 (0.84–1.05) | 1.12 (0.95–1.32) |
| **IgG < 1120 mg/dL** | 1.00 (0.91–1.11) | 0.92 (0.79–1.08) |
| **IgA < 187 mg/dL** | 1.02 (0.92–1.13) | 0.85 (0.71–1.00) |

Across both 10-year models, censoring for hyperparathyroidism and for schizophrenia, the associations between low Ig levels and subsequent disease risk were not statistically significant, as all confidence intervals for the Ig -related hazard ratios crossed 1. For hyperparathyroidism, age and female sex showed significant associations, while for schizophrenia, younger age and male sex were significantly associated with increased risk. Overall, these findings suggest that, within the first decade of follow-up, reduced IgM, IgG, or IgA levels do not independently predict either outcome after adjusting for age and sex.

**Sub-analysis: number of patients**

To characterize the distribution of CLL events across follow-up periods and Ig categories, we examined patient and measurement-level statistics across different analytical approaches and time windows.

Patient-Level Analysis (First Immunoglobulin Measurement Only):

The primary Cox regression analysis included 1,571 patients who developed CLL within 10 years of their first Ig measurement (out of 294,712 total patients, representing 0.53% cumulative incidence). Of these 1,571 CLL cases, 1,397 patients (88.9%) had at least one of the three Ig classes (IgA, IgM, or IgG) in the low-normal range (below CLL cohort median thresholds) during the 10 years before the diagnosis of CLL. This high proportion demonstrates that the vast majority of individuals who subsequently developed CLL exhibited detectable Ig alterations within normal ranges years before diagnosis.

Examining temporal distribution, when restricting CLL diagnoses occurring 10 to 2 years after measurement (excluding the two last years), 1,304 patients (83.0% of total events) remained, with 1,167 (89.5%) having low-normal Ig. When further restricting to diagnoses 5-10 years before measurement, 1,083 patients (68.9% of total events) remained, demonstrating that a substantial proportion of associations reflect long-term risk rather than imminent disease. This extended temporal reach argues against pure reverse causation from symptomatic prodrome influencing measurement ascertainment.

Measurement-Level Analysis (All Available Immunoglobulin Measurements):

When including all available Ig measurements per patient rather than only the first, 4,083 blood samples preceded a CLL diagnosis within 10 years. Of these, 3,819 samples (93.5%) exhibited at least one low-normal Ig value, even higher than the first-measurement-only analysis, suggesting progressive Ig decline as individuals approach clinical diagnosis. In the 2-10 year window, 3,653 samples (89.5% of total) remained, and in the 5-10 year window, 3,081 samples (75.4%) remained, confirming robust long-term associations.

The consistently high proportion of low-normal Ig across all time windows (88-94%) reinforces that Ig alterations are highly prevalent in the preclinical phase of CLL, validating their potential utility as screening biomarkers.

**Sub-analysis: IgM**

The observation that IgM levels still demonstrate significant predictive value when analyzed as low-normal (HR 1.77) rather than pathologically low suggests that traditional dichotomous categorization (normal vs. abnormal) may have obscured the capacity of Ig measurements to detect very early disease stages. Notably, while the median IgM level was lower in the CLL cohort (70.50 mg/dL) compared to controls (98.50 mg/dL), the mean IgM was higher (166.81 vs 122.59 mg/dL) with a large standard deviation (291.18). This suggests that while the central tendency for pre-CLL patients is toward lower IgM, a subset of individuals may have high-value outliers. It may correspond to CLL with an IgM monoclonal gammopathy identifies a high-risk patient subgroup with poorer prognosis. This subgroup is linked to specific genetic markers like unmutated IGHV and TP53 aberrations [1].

[1] Yan Y, Yuan B, Qiu T, et al. Monoclonal gammopathy defines distinct clinical subsets in Chronic Lymphocytic Leukemia across therapeutic eras. Blood Advances 2025:bloodadvances.2025017057. https://doi.org/10.1182/bloodadvances.2025017057.

Reconciling Kaplan-Meier and Cox Regression Findings for IgM by Sex

The Kaplan-Meier curves and Cox regression analyses reveal apparently discordant sex-specific patterns for IgM that require careful interpretation. In the KM curves, low IgM shows similar or slightly greater absolute risk elevation in females compared to males (10-year cumulative incidence: 0.85% in females vs 1.06% in males with low IgM). However, the sex-stratified Cox regression demonstrates that low IgM confers a stronger relative hazard in females (HR 2.06, CI 1.72-2.47) than in males (HR 1.53, CI 1.29-1.81).

This apparent paradox is explained by differential baseline CLL incidence between sexes. The KM curves show absolute risk, where males have higher baseline CLL rates regardless of Ig status (control male risk: 0.25% vs female: 0.17%). Therefore, adding low IgM to an already higher male baseline produces a smaller multiplicative effect (HR 1.53) despite higher absolute risk (1.06%). Conversely, in females with lower baseline risk, low IgM represents a more selective marker requiring a larger multiplicative effect (HR 2.06) to reach a slightly lower absolute risk (0.85%).

The relative risk calculation comparing males to females within the low IgM group (RR 1.24, CI 1.05-1.47) reflects the absolute difference visible in KM curves, confirming males have higher absolute risk. However, this does not contradict the sex-specific hazard ratios, which measure the effect of low IgM relative to each sex's own baseline.

**Sub-analysis : Dual mechanism analysis**

Formal Testing of Dual-Mechanism Hypothesis Through Lymphocyte- Ig Interaction Analysis

**Methods:** To formally test whether Ig associations operate through dual mechanisms (constitutional susceptibility and tumor burden), we performed 2×2 stratification by baseline lymphocyte count and Ig level. Patients were categorized into normal (0.8-3.6 × 10⁹/L) versus elevated-normal (3.0-4.8 × 10⁹/L) lymphocyte counts, crossed with low versus high Ig levels. Cox regression models adjusted for age and sex were performed within each stratum, with the low-lymphocyte/high- Ig group as reference.

**Results:** IgA demonstrated near-multiplicative interaction (observed HR 2.51 vs predicted 2.29, ratio 1.10), consistent with independent constitutional (HR 1.85 in low-lymphocyte patients) and tumor-burden (HR 1.24 in high- Ig patients) effects. The persistence of IgA associations even in patients with normal lymphocyte counts (HR 1.85), where substantial MBL is unlikely, supports a constitutional susceptibility mechanism operating independently of clonal burden.

IgG showed sub-multiplicative interaction (observed 1.50 vs predicted 1.68, ratio 0.89), suggesting partial mechanistic overlap. The attenuated effect in normal-lymphocyte patients (HR 1.10, 95% CI 0.86-1.42 crossing unity) indicates IgG primarily reflects tumor-mediated suppression rather than constitutional susceptibility.

IgM demonstrated super-multiplicative interaction (observed 1.78 vs predicted 1.14, ratio 1.56), suggesting potential synergistic effects. However, the very weak associations in both single-factor groups (HR 1.11 and 1.03) with wide confidence intervals indicate limited independent effects, with the elevated risk concentrated in patients with both high lymphocytes and low IgM.

**Interpretation:** These patterns support a dual-mechanism model wherein IgA captures both constitutional susceptibility (detectable even without lymphocytosis) and tumor-mediated suppression (amplified by elevated lymphocyte counts), whereas IgM and IgG function primarily as lymphocyte-dependent markers of prevalent MBL. The differential behavior across Ig classes provides mechanistic insights into early CLL pathogenesis and may inform risk stratification strategies.

**Tables: Stratified Analysis by Lymphocyte Count and Immunoglobulin Levels**

**2×2 Stratification Testing Dual-Mechanism Hypothesis (0-10 years follow-up)**

**A. IgA Stratification**

| **Group** | **Lymphocyte Count** | **IgA Level** | **HR (95% CI)** | **Interpretation** |
| --- | --- | --- | --- | --- |
| 1 (ref) | 0.8-3.6 × 10⁹/L | ≥187 mg/dL | 1.00 | Reference |
| 2 | 0.8-3.6 × 10⁹/L | <187 mg/dL | 1.85 (1.45-2.37) | Constitutional effect |
| 3 | 3.0-4.8 × 10⁹/L | ≥187 mg/dL | 1.24 (0.56-2.77) | Tumor burden effect |
| 4 | 3.0-4.8 × 10⁹/L | <187 mg/dL | 2.51 (1.76-3.58) | Combined effect |

**Multiplicative prediction:** 1.85 × 1.24 = 2.29
**Observed Group 4:** 2.51
**Ratio:** 2.51/2.29 = 1.10 (consistent with multiplicative interaction)

**B. IgG Stratification**

| **Group** | **Lymphocyte Count** | **IgG Level** | **HR (95% CI)** | **Interpretation** |
| --- | --- | --- | --- | --- |
| 1 (ref) | 0.8-3.6 × 10⁹/L | ≥1120 mg/dL | 1.00 | Reference |
| 2 | 0.8-3.6 × 10⁹/L | <1120 mg/dL | 1.10 (0.86-1.42) | Constitutional effect |
| 3 | 3.0-4.8 × 10⁹/L | ≥1120 mg/dL | 1.53 (0.69-3.43) | Tumor burden effect |
| 4 | 3.0-4.8 × 10⁹/L | <1120 mg/dL | 1.50 (1.04-2.17) | Combined effect |

**Multiplicative prediction:** 1.10 × 1.53 = 1.68
**Observed Group 4:** 1.50
**Ratio:** 1.50/1.68 = 0.89 (sub-multiplicative, partial overlap)

**C. IgM Stratification**

| **Group** | **Lymphocyte Count** | **IgM Level** | **HR (95% CI)** | **Interpretation** |
| --- | --- | --- | --- | --- |
| 1 (ref) | 0.8-3.6 × 10⁹/L | ≥70.5 mg/dL | 1.00 | Reference |
| 2 | 0.8-3.6 × 10⁹/L | <70.5 mg/dL | 1.11 (0.86-1.42) | Constitutional effect |
| 3 | 3.0-4.8 × 10⁹/L | ≥70.5 mg/dL | 1.03 (0.43-2.48) | Tumor burden effect |
| 4 | 3.0-4.8 × 10⁹/L | <70.5 mg/dL | 1.78 (1.25-2.52) | Combined effect |

**Multiplicative prediction:** 1.11 × 1.03 = 1.14
**Observed Group 4:** 1.78
**Ratio:** 1.78/1.14 = 1.56 (super-multiplicative, synergistic)

**Sensitivity Analysis with Additional Clinical Covariates**

**Expanded Confounder Adjustment**

To comprehensively assess whether Ig -CLL associations reflect confounding by clinical conditions that alter Ig levels, we performed sensitivity analyses with expanded covariate adjustment beyond age and sex (binary, up to 1 year before).

Detailed covariate definitions:

**Immunosuppressive drugs (non-biologic):** Any of: SIROLIMUS, EVEROLIMUS, CHLORAMBUCIL, CICLOSPORINE, CYCLOPHOSPHAMIDE, LEFLUNOMIDE, CICLOSPORIN, MYCOPHENOLIC ACID, AZATHIOPRINE, TACROLIMUS, METHOTREXATE.

**Biologic immunosuppressive therapy:** Any of: BELIMUMAB, ABATACEPT, CERTOLIZUMAB, GOLIMUMAB, SECUKINUMAB, USTEKINUMAB, TOCILIZUMAB, ETANERCEPT, INFLIXIMAB, ADALIMUMAB, excluding anti-CD20 agents (analyzed separately).

**Immunodepressed state (composite):** Any of: solid organ transplant (ICD-9: V42.x, 996.8x), bone marrow transplant, active chemotherapy for non-hematologic malignancy (ICD-9: V58.11), or any immunosuppressive drug, or use of chronic steroid (V58.65).

**Chronic infection:** 595.2* Other Chronic Cystitis, 473* Chronic Sinusitis, 070.54* Chr. Hepatitis C, 070.44* Chronic Hepatitis C, 070.32* Viral Hepatitis B, 070.22* Viral Hepatitis B+H, 042* AIDS, 031.0* Pulmonary Diseases (Mycobacterium), 011* Pulmonary Tuberculosis.

**Autoimmune disease:** Any of: 720* Ankylosing Spondylitis, 714* Rheumatoid Arthritis, 710* Diffuse Diseases of Connective Tissue, 446* Polyarteritis Nodosa, 287.31* Immune Thrombocytopenia, 283.0* Autoimmune Hemolytic Anemia, 710.4* Polymyositis, 710.3* Dermatomyositis, 710.2* Sicca Syndrome, 710.1* Systemic Sclerosis, 710.0* Systemic Lupus Erythematosus, 696.0* Psoriatic Arthropathy, 694.5* Pemphigoid, 694.4* Pemphigus, 579.0* Celiac Disease, 556* Idiopathic Proctocolitis, 555* Regional Enteritis, 446.5* Giant Cell Arteritis, 446.4* Wegener's Granulomatosis, 358.0 Myasthenia Gravis, 340* Multiple Sclerosis, 245.2* Chronic Lymphocytic Thyroiditis

**Rituximab exposure:** Anti-CD20 monoclonal antibody prescription recorded before index date. Analyzed separately due to direct B-cell depletion mechanism distinct from other immunosuppression.

*Statistical approach:* We compared hazard ratios from the primary parsimonious model (age + sex + Igs) to an extended model including all clinical covariates. Non-biologic immunosuppressive drugs and biologic therapy were entered as separate indicators to assess dose-response relationships and distinguish mechanisms.

**Supplementary Results:** Robustness to Comprehensive Confounder Adjustment

Adjustment for comprehensive clinical covariates produced negligible changes in Ig -CLL associations (Supplement Table):

IgA: Primary HR 2.62 → Adjusted HR 2.61 (−0.4% change; 95% CI 2.32-2.94)
IgM: Primary HR 1.77 → Adjusted HR 1.76 (−0.6% change; 95% CI 1.57-1.99)
IgG: Primary HR 1.43 → Adjusted HR 1.43 (0% change; 95% CI 1.26-1.61)

All Ig associations retained statistical significance with confidence intervals overlapping primary model estimates. The analyses restricted to 2-10 years (excluding the 2 years immediately preceding diagnosis to minimize reverse causation) showed similar stability: IgA HR 2.01 (adjusted) vs 2.02 (primary), IgM HR 1.48 vs 1.48, IgG HR 1.35 vs 1.35.

The null or inverse associations for conditions that suppress Ig production through distinct mechanisms (autoimmune disease via dysregulation, chronic infections via consumption, immunosuppressive drugs via therapeutic suppression) provide strong evidence against confounding by indication. If low Ig simply reflected underlying disease burden or treatment effects, these conditions should show positive associations with CLL, which they do not.

Rituximab interpretation: The elevated CLL risk among rituximab-exposed patients likely reflects treatment of underlying lymphoproliferative disorders (marginal zone lymphoma, lymphoplasmacytic lymphoma) that share pathogenic features with CLL, or severe autoimmune conditions with genetic susceptibility overlapping CLL risk factors. Rituximab directly depletes B-cells and suppresses Ig synthesis, yet this small subset does not drive overall Ig associations: excluding rituximab-exposed patients yielded identical hazard ratios (IgA HR 2.62, IgM HR 1.78, IgG HR 1.44; data not shown).

Inverse associations with immunosuppression: The unexpected inverse or null associations between immunosuppressed states and CLL warrant mechanistic consideration. Possible explanations include: (1) competing mortality — immunosuppressed patients face elevated risks of infection, cardiovascular disease, and non-hematologic malignancies, reducing survival to CLL diagnosis; (2) surveillance bias — intensive monitoring may detect and manage other complications, paradoxically preventing MBL progression; (3) biological protection — chronic immunosuppression may inhibit clonal B-cell expansion through mechanisms distinct from Ig suppression.

**Conclusion:** The negligible impact of adjusting for comprehensive clinical covariates, combined with null/inverse associations for conditions that alter Ig levels through diverse mechanisms, strongly argues that Ig -CLL relationships reflect intrinsic biological associations rather than confounding by indication, underlying disease states, or iatrogenic immunosuppression.

**Supplement Table Comprehensive Sensitivity Analysis with Clinical Covariates**

Multivariable Cox proportional hazards model with expanded covariate adjustment (N=294,712 patients, 1,571 CLL events)

| **Feature** | **0–10 Years HR (95% CI)** | **2–10 Years HR (95% CI)** | **Comparison to Primary Model*** |
| --- | --- | --- | --- |
| Age (per year) | 1.04 (1.04–1.05) | 1.04 (1.03–1.05) | No change |
| Male sex | 1.55 (1.38–1.75) | 1.44 (1.23–1.68) | No change (1.55 → 1.55) |
| **IgM <70.5 mg/dL** | **1.76 (1.57–1.99)** | **1.48 (1.26–1.73)** | **No change (1.77 → 1.76, −0.6%)** |
| **IgG <1120 mg/dL** | **1.43 (1.26–1.61)** | **1.35 (1.14–1.59)** | **No change (1.43 → 1.43, 0%)** |
| **IgA <187 mg/dL** | **2.61 (2.32–2.94)** | **2.01 (1.71–2.36)** | **No change (2.62 → 2.61, −0.4%)** |
| Immunosuppressive drugs (non-biologic)† | 1.00 (0.52–1.95) | 1.34 (0.59–3.03) | — |
| Biologic immunosuppressive therapy‡ | Not estimable§ | Not estimable§ | — |
| Immunodepressed state¶ | 0.85 (0.59–1.24) | 0.89 (0.54–1.46) | — |
| Chronic infection‖ | 1.13 (0.54–2.37) | 0.56 (0.14–2.24) | — |
| Autoimmune disease** | 0.77 (0.50–1.19) | 0.63 (0.34–1.19) | — |
| Rituximab exposure†† | 2.04 (1.12–3.70) | 1.71 (0.71–4.15) | — |
| *Primary model: Age + Sex + IgM + IgG + IgA (IgA HR 2.62, IgM HR 1.77, IgG HR 1.43) †conventional immunosuppressants (methotrexate, azathioprine, mycophenolate, cyclosporine, tacrolimus) ‡Anti-TNF, anti-IL (excluding anti-CD20) §Too few events in biologic-exposed group for stable estimation ¶Composite: transplant status, active chemotherapy, or any of the above ‖HIV, chronic viral hepatitis (B/C), chronic bacterial infections  **Rheumatoid arthritis, SLE, IBD, Sjögren's, systemic sclerosis, autoimmune thyroid disease, type 1 diabetes, celiac disease, MS, myasthenia gravis ††Anti-CD20 monoclonal antibody exposure before index date (rituximab; n=142 patients, 0.05% of cohort) | | | |

**Interpretation:** Ig -CLL associations showed negligible attenuation (<1% change in hazard ratios) after adjusting for comprehensive clinical covariates. All Ig associations retained statistical significance (p<0.001) with overlapping confidence intervals to primary models. Notably, autoimmune disease (HR 0.77, 95% CI 0.50-1.19), immunosuppressed state (HR 0.85, 95% CI 0.59-1.24), and chronic infection (HR 1.13, 95% CI 0.54-2.37) showed null or inverse associations, arguing strongly against confounding by these conditions. Rituximab exposure showed elevated risk (HR 2.04, 95% CI 1.12-3.70), likely reflecting treatment of underlying lymphoproliferative disorders rather than drug-induced immunosuppression.

**Sensitivity Analysis Restricted to Ages 40-70 Years**

Cox proportional hazards models in younger cohort with lower competing mortality risk (N=200,503 patients, 831 CLL events)

**Competing Risk Sensitivity Analysis**

To address potential competing risk bias from non-CLL mortality, we performed sensitivity analysis restricted to patients aged 40-70 years at Ig measurement (excluding those aged 70-80 years), where mortality from cardiovascular disease, non-hematologic malignancies, and other competing causes is substantially lower. This restriction reduces the 10-year all-cause mortality rate from approximately 15-20% in the 70-80 age group to 5-10% in the 40-70 age group, minimizing the probability that patients die before developing clinically apparent CLL.

While formal Fine-Gray competing risk models were not available in our analytical platform, age restriction provides a valid alternative approach to assess whether competing mortality artificially inflates observed associations. If Ig -CLL relationships were artifacts of competing risks (e.g., low Ig → death before CLL diagnosis → spurious "protective" effect that we misinterpret as risk when absent), we would expect attenuated or null associations in the younger cohort where competing events are rare. Conversely, consistent or stronger associations in the age-restricted cohort support genuine biological relationships independent of competing mortality.

**Supplementary Results: Robustness in Low Competing-Risk Population**

Restriction to ages 40-70 years (N=200,503, 831 CLL events) yielded consistent or stronger Ig -CLL associations compared to the full cohort (Supplement Table X):

- **IgA <187 mg/dL:** HR 2.42 (95% CI 2.08-2.82) vs 2.62 in full cohort
- **IgM <70.5 mg/dL:** HR 2.03 (95% CI 1.74-2.36) vs 1.77 in full cohort
- **IgG <1120 mg/dL:** HR 1.42 (95% CI 1.22-1.66) vs 1.43 in full cohort

All associations remained highly statistically significant (p<0.001) with overlapping 95% confidence intervals to full cohort estimates. The modestly stronger IgM association in younger patients (HR 2.03 vs 1.77) may reflect age-dependent differences in MBL biology or reduced confounding by age-related comorbidities that can suppress Ig independently of CLL pathogenesis.

The consistency of findings across age strata with different competing risk profiles provides strong evidence that observed associations reflect genuine Ig -CLL relationships rather than artifacts of selective survival or informative censoring. Patients with low Ig in the younger cohort—where the probability of surviving to CLL diagnosis is high—demonstrate elevated CLL risk at magnitudes similar to or greater than the overall population, arguing against competing mortality as an alternative explanation.

**Interpretation:** The robustness of Ig associations in a population with minimal competing mortality risk addresses a key limitation of standard Cox regression in the presence of competing events. While Fine-Gray models would provide formal statistical testing of subdistribution hazards, the biological interpretation remains similar: low Ig levels identify individuals at elevated risk of developing CLL among those who survive long enough for diagnosis to occur. Our age-restricted analyses demonstrate this relationship operates even when competing mortality is rare, supporting causal inference.

| **Feature** | **0–10 years HR (95% CI)** | **2–10 years HR (95% CI)** | **Comparison to Full Cohort (40-80y)*** |
| --- | --- | --- | --- |
| Age (per year) | 1.06 (1.05–1.07) | 1.06 (1.05–1.07) | Stronger (1.04 → 1.06) |
| Male sex | 1.56 (1.34–1.81) | 1.47 (1.20–1.79) | Similar (1.55 → 1.56) |
| **IgM <70.5 mg/dL** | **2.03 (1.74–2.36)** | **1.58 (1.29–1.93)** | **Stronger (1.77 → 2.03)** |
| **IgG <1120 mg/dL** | **1.42 (1.22–1.66)** | **1.24 (1.01–1.52)** | **Similar (1.43 → 1.42)** |
| **IgA <187 mg/dL** | **2.42 (2.08–2.82)** | **1.90 (1.55–2.32)** | **Similar (2.62 → 2.42)** |

*Full cohort (ages 40-80): IgA HR 2.62, IgM HR 1.77, IgG HR 1.43

**Interpretation:** Restriction to ages 40-70 years, where competing mortality risk is substantially lower, yielded consistent or stronger Ig -CLL associations compared to the full cohort. All associations remained statistically significant with overlapping confidence intervals, indicating that competing risks do not spuriously create the observed relationships. The slightly stronger IgM association (HR 2.03 vs 1.77) in younger patients may reflect reduced confounding by age-related comorbidities or differential MBL biology across age strata.
