# Supplementary material for: Low-normal immunoglobulin suggests monoclonal B-cell lymphocytosis and constitutional CLL susceptibility: a dual-mechanism analysis
Source: Leukemia. 2026 Jun 10;40(8):1668–75. doi: 10.1038/s41375-026-02987-2 (PMC13421330; doi:10.1038/s41375-026-02987-2)
Supplement: Supplementary file 2 — supplement 1 [file 41375_2026_2987_MOESM2_ESM.docx]

**Supplement (1)**

**Complement information about the study**

**Complement information about methods and statistics**

The study was based on the STROBE guidelines. This retrospective quantitative observational study aimed to evaluate the association between immunoglobulin (Ig) levels and the development of CLL. A quantitative study design was employed to enable robust statistical assessment of time-to-event outcomes through survival analysis methods. This approach is particularly suited for the evaluation of real-world data routinely generated in clinical practice, as it facilitates the investigation of relationships between clinical biomarkers and patient outcomes while accounting for potential confounding factors.

To enhance diagnostic validity, absolute lymphocyte counts, and relative lymphocyte percentages were cross-checked against population reference values around the diagnosis of CLL. Confirmation of elevated lymphocyte levels in temporal proximity to the recorded CLL diagnosis was required. We also evaluate survival, knowing the poorer overall survival of CLL patients compared to the general population. Because this was a retrospective analysis that included all available data in the health system, a formal a priori power calculation was not required. However, the large number of observed events (N = 1,571) provides substantial statistical power to detect the effect sizes reported in this study.

The final analytical cohort was restricted to 294,712 patients who had complete data for all variables included in the Cox regression models (IgG, IgA, IgM, age, and sex). Therefore, no imputation for missing data was required for the primary analysis. Data quality was ensured by excluding individuals born outside the country or those who discontinued membership in the health system, thereby preventing incomplete follow-up and loss to documentation.

The proportional hazards assumption was evaluated using a piecewise Cox proportional hazards approach to ensure model stability over time. In addition, we performed a Cox regression restricted to the period from 2 to 10 years after the index date to minimize the risk of reverse causation and outcome-driven bias. To confirm that patients showed no early indications of CLL at the index date, another cox regression with Ig levels analyzed only when a same-day lymphocyte count below 4.8 × 10⁹/L was documented.

Cohort Entry and Follow-Up: To strictly avoid immortal time bias, this study did not use a fixed entry date for all participants. Instead, we employed a measurement-anchored design. A participant's entry into the risk set (T0) was strictly set to the date of their first available Ig test performed between the ages of 40 and 80. No person-time prior to this measurement was included in the analysis. For example, a patient included in the 2004 database extract who underwent their first Ig test in 2010 contributed person-time to the analysis only from 2010 onwards. Incident CLL diagnoses were only considered if they occurred after this index date.

**Control quality of sample**

For descriptive analyses comparing baseline characteristics, the 'Control' group consists of randomly age (40 – 80 years) and sex matched patients (155,700 patients) without diagnosis of CLL, index date also randomal.

1. *Lymphocyte at diagnosis comparison between CLL and control*

| **Feature** | **Diagnosis – Case Mean (SD)** | **Diagnosis – Case Median** | **Diagnosis – Control Mean (SD)** | **Diagnosis – Control Median** |
| --- | --- | --- | --- | --- |
| **Lym (abs)** | 12.29 (19.44) | 7.10 | 2.17 (1.03) | 2.09 |
| **Lym (%)** | 53.98 (18.75) | 56.00 | 30.70 (8.47) | 30.50 |

At diagnosis, lymphocyte levels were significantly higher in cases than in controls.

1. *Lymphocyte absolute level comparison between CLL and control*


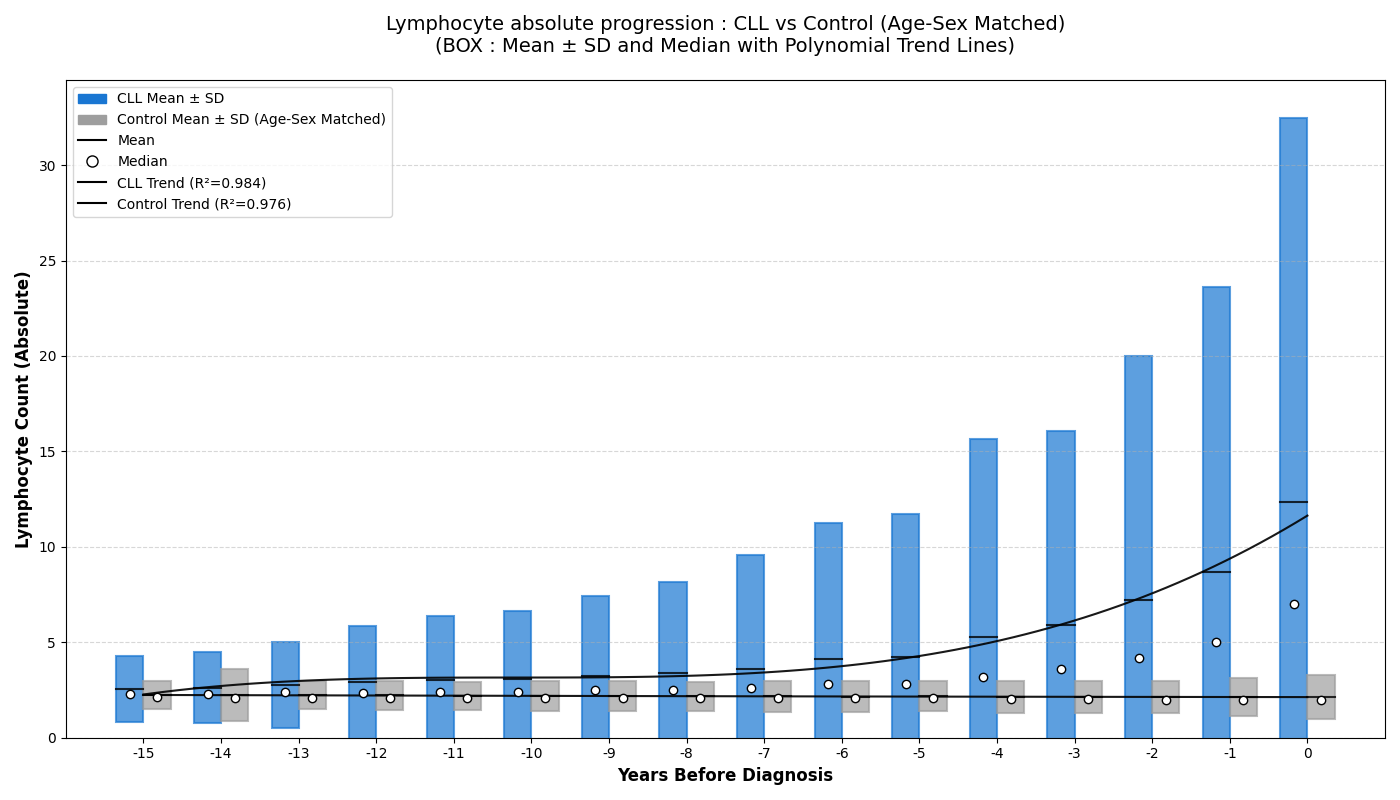


As seen, there is a higher level of lymphocyte close to the diagnosis

1. *Survival curves (up to 15 years)*


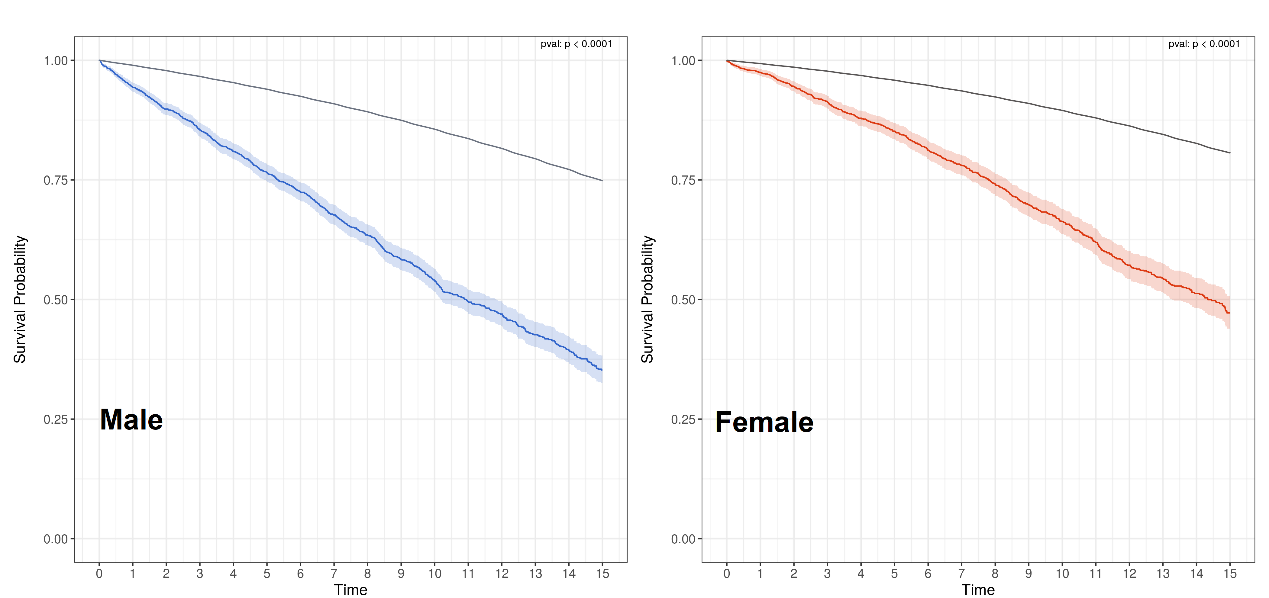


As known from the literature, patients with CLL have a poorer survival rate.

**Diagram of eligibility for cox regression and Kaplan-Meier curves**

**Chart flow**

**Patients with IgG sample (excluded if born in another country or quit CHS**)

683,623 patients

**Identification**

**IgG level between 40 – 80 years** (excluded: 375,958 patients)

307,665 patients

**Exclusion**

**Exclusion if IgA or IgM levels were not reported on the same day** (Excluded: 12,953 patients)

294,712 patients

(For the cox regression)

**Level of IgA less than**

90,771 patients

**Level of IgA more than**

203,941 patients

**Level of IgG less than**

130,176 patients

**Level of IgG more than**

164,536 patients

**Level of IgM less than**

98,101 patients

**Level of IgM more than**

196,611

(For the Kaplan-Meier curves)

**Included**

**Cox regression: Ig as continuous variables (10 years)**

| **Feature** | **HR (95% CI: Lower–Upper)** |
| --- | --- |
| **Age** | **1.043792912 (1.03907906–1.048528149)** |
| **Male sex** | **1.795382864 (1.641245606–1.963995892)** |
| **IgM** | **0.998477244 (0.997899861–0.999054961)** |
| **IgG** | **0.999315368 (0.999161956–0.999468803)** |
| **IgA** | **0.993773036 (0.993235358–0.994311005)** |

**Continuous Immunoglobulin Associations with CLL Risk (0-10 years)**

| **Immunoglobulin** | **HR per 100 mg/dL Decrease (95% CI)** |
| --- | --- |
| **IgA** | **1.87 (1.76-1.99)** |
| **IgM** | **1.16 (1.10-1.23)** |
| **IgG** | **1.07 (1.06-1.09)** |

When modeled as continuous variables, each 100 mg/dL decrease in Ig level was associated with increased CLL risk: IgA HR 1.87 (95% CI 1.76-1.99), IgM HR 1.16 (95% CI 1.10-1.23), and IgG HR 1.07 (95% CI 1.06-1.09). These continuous associations demonstrate dose-dependent relationships across the entire normal range without requiring arbitrary categorization.

**Tendency of immunoglobulin up to 15 years before diagnosis**

Box plots show mean ± SD (boxes) and median (circles) at annual intervals up to 14 years before CLL diagnosis. Polynomial trend lines fitted to capture non-linear temporal dynamics (CLL: blue line; Control: black line). Generate with Python 3.11 based on data extract from Wiser.

**(A) IgA** demonstrates progressive linear decline in CLL (R²=0.728) with early divergence from controls (R²=0.439). The high CLL R² indicates uniform disease-driven suppression across patients, while lower control R² reflects normal variability, supporting constitutional susceptibility and consistent gradual immunosuppression as a hallmark CLL feature.

**
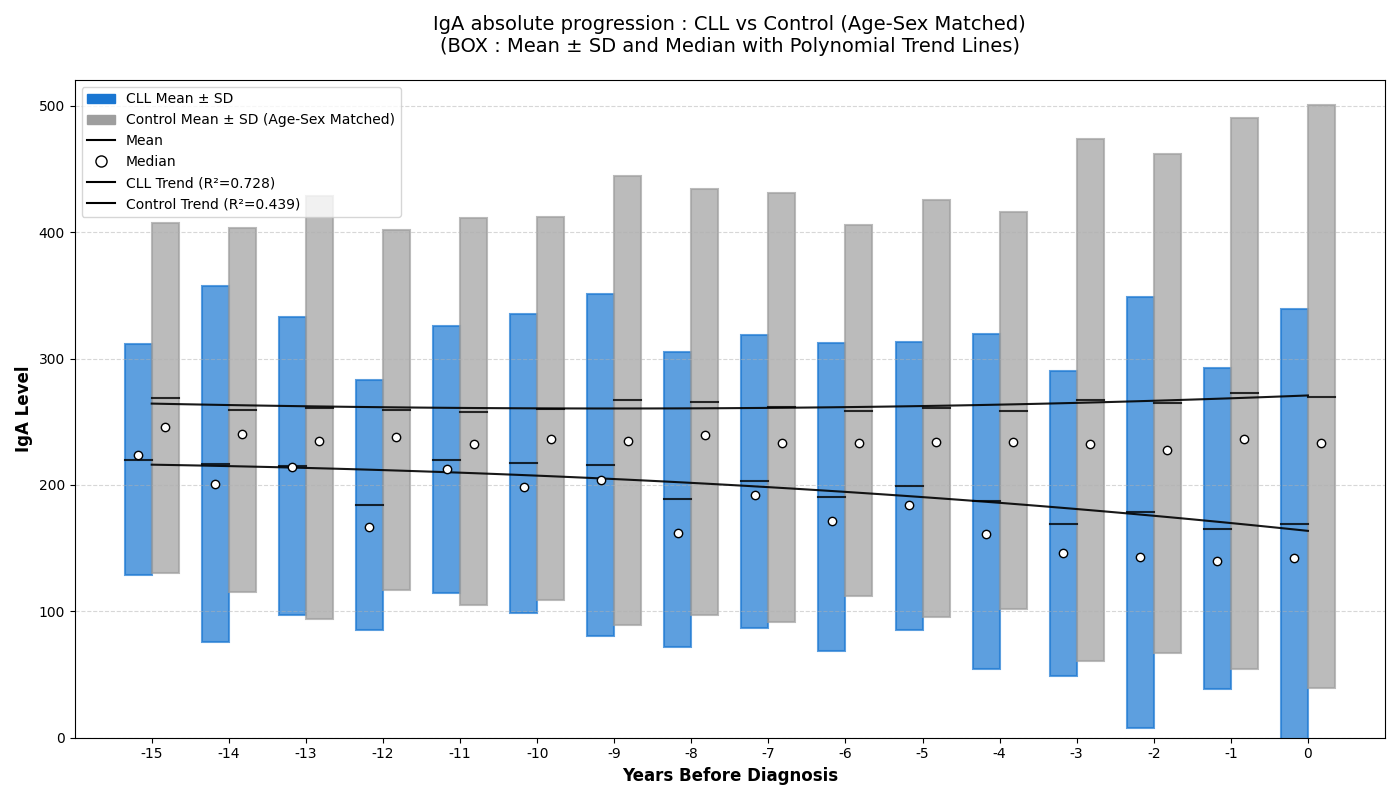
**

**(B) IgG** demonstrates stable values until -5 years followed by accelerated decline at diagnosis (R²=0.547), consistent with late-stage tumor burden effects.

**
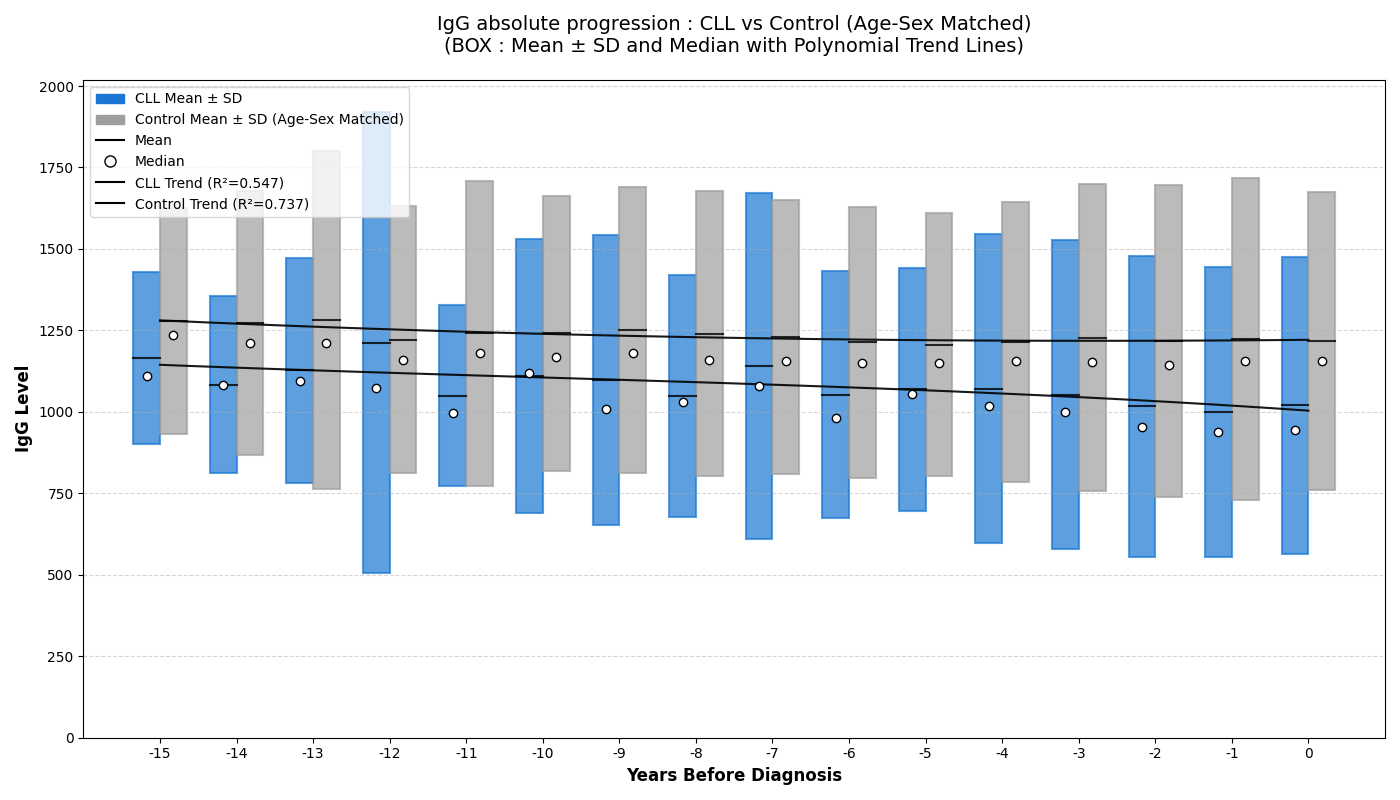
**

**(C) IgM** shows persistent median suppression (53-118 mg/dL vs control 83-109 mg/dL) throughout 15 years. Discordance between low median and elevated mean with large SD reflects a bimodal distribution: most patients exhibit sustained IgM suppression, while ~10-15% seems to develop monoclonal paraproteins that inflate the mean (R²=0.567).

**
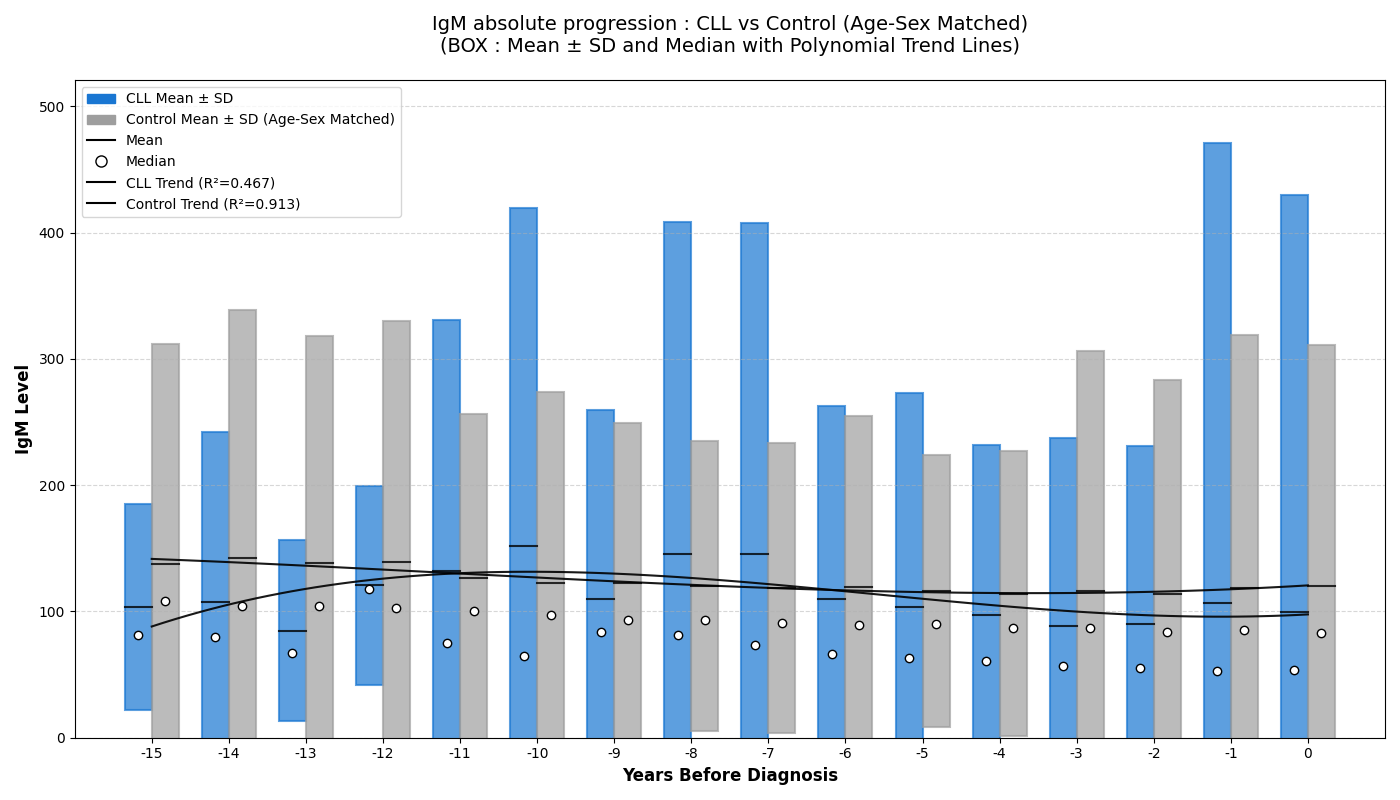
**

**Characteristics of the Population of Kaplan Meir Curves at index date (entry in the study)**

Comparison of Laboratory and Clinical Characteristics by Ig Level at Index Date

**A. IgM ≤70.5 mg/dL vs >70.5 mg/dL**

| **Feature** | **IgM ≤70.5 mg/dL (Mean ± SD)** | **IgM >70.5 mg/dL (Mean ± SD)** | **IgM ≤70.5 mg/dL (Median)** | **IgM >70.5 mg/dL (Median)** |
| --- | --- | --- | --- | --- |
| **Age (years)** | 64.27 ± 10.37 | 60.39 ± 11.19 | 65.89 | 60.97 |
| **Lymphocytes (×10⁹/L)** | 2.26 ± 4.14 | 2.05 ± 1.66 | 1.90 | 1.90 |
| **IgM (mg/dL)** | 48.38 ± 14.57 | 149.79 ± 155.59 | 50.00 | 119.00 |
| **IgG (mg/dL)** | 1145.69 ± 444.28 | 1249.77 ± 374.90 | 1090.00 | 1199.00 |
| **IgA (mg/dL)** | 263.15 ± 220.09 | 264.63 ± 136.33 | 232.00 | 240.00 |

**B. IgG ≤1120 mg/dL vs >1120 mg/dL**

| **Feature** | **IgG ≤1120 mg/dL (Mean ± SD)** | **IgG >1120 mg/dL (Mean ± SD)** | **IgG ≤1120 mg/dL (Median)** | **IgG >1120 mg/dL (Median)** |
| --- | --- | --- | --- | --- |
| **Age (years)** | 62.96 ± 10.79 | 61.16 ± 11.21 | 64.35 | 62.06 |
| **Lymphocytes (×10⁹/L)** | 2.20 ± 3.90 | 2.09 ± 2.57 | 1.87 | 1.90 |
| **IgM (mg/dL)** | 108.81 ± 165.80 | 122.48 ± 115.92 | 81.80 | 99.80 |
| **IgG (mg/dL)** | 936.60 ± 147.80 | 1429.71 ± 386.60 | 970.00 | 1330.00 |
| **IgA (mg/dL)** | 226.70 ± 185.27 | 293.23 ± 145.54 | 202.00 | 267.00 |

1. **IgA ≤187 mg/dL vs >187 mg/dL**

| **Feature** | **IgA ≤187 mg/dL (Mean ± SD)** | **IgA >187 mg/dL (Mean ± SD)** | **IgA ≤187 mg/dL (Median)** | **IgA >187 mg/dL (Median)** |
| --- | --- | --- | --- | --- |
| **Age (years)** | 61.73 ± 11.15 | 61.66 ± 11.05 | 62.83 | 62.71 |
| **Lymphocytes (×10⁹/L)** | 2.25 ± 4.32 | 2.06 ± 1.62 | 1.81 | 1.90 |
| **IgM (mg/dL)** | 121.50 ± 196.06 | 113.53 ± 97.75 | 88.00 | 93.00 |
| **IgG (mg/dL)** | 1088.32 ± 441.27 | 1271.70 ± 369.93 | 1035.00 | 1220.00 |
| **IgA (mg/dL)** | 137.53 ± 36.65 | 320.70 ± 174.12 | 144.90 | 283.00 |

Kaplan-Meir absolute risk with IC95 at 10 years (control and groups)

| **Feature** | **Male** | **Female** |
| --- | --- | --- |
| **Control** | 0.0025 (0.0011–0.0039) | 0.0017 (0.0008–0.0027) |
| **IgG > 1120 mg/dL** | 0.0056 (0.0049–0.0064) | 0.0035 (0.0030–0.0040) |
| **IgG < 1120 mg/dL** | 0.0106 (0.0095–0.0116) | 0.0067 (0.0060–0.0074) |
| **IgM > 70.5 mg/dL** | 0.0057 (0.0050–0.0064) | 0.0036 (0.0031–0.0040) |
| **IgM < 70.5 mg/dL** | 0.0106 (0.0094–0.0117) | 0.0085 (0.0075–0.0096) |
| **IgA > 187 mg/dL** | 0.0051 (0.0045–0.0057) | 0.0032 (0.0028–0.0036) |
| **IgA < 187 mg/dL** | 0.0145 (0.0129–0.0161) | 0.0083 (0.0073–0.0093) |

Complete table of blood samples at index date: CLL vs random control

| **Feature** | **CLL Median** | **Control Median** | **CLL Mean (SD)** | **Control Mean (SD)** | **p-value*** |
| --- | --- | --- | --- | --- | --- |
| **IgM mg/dL** | 70.50 | 98.50 | 166.81 (±291.18) | 122.59 (±74.48) | <0.01 |
| **IgG mg/dL** | 1120.00 | 1210.00 | 1136.18 (±442.77) | 1279.94 (±437.07) | <0.01 |
| **IgA mg/dL** | 187.50 | 266.00 | 216.14 (±128.66) | 268.02 ± (115.60) | <0.01 |
| **Lym (abs)** | 2.40 | 2.10 | 3.08 (±3.58) | 2.20 (±0.72) | <0.01 |
| **Lym (%)** | 33.10 | 31.00 | 34.31 (±11.31) | 31.06 (±8.44) | <0.01 |
| **IgE kU/L** | 76.50 | 63.00 | 334.90 (±583.66) | 257.56 (±758.61) | <0.01 |
| **Globulin g/dL** | 2.97 | 3.01 | 2.965 (±0.42) | 3.066 (±0.41) | <0.01 |
| **Baso (abs)** | 0.03 | 0.02 | 0.044 (±0.062) | 0.034 (±0.042) | <0.01 |
| **Eos (abs)** | 0.20 | 0.20 | 0.221 (±0.17) | 0.210 (±0.17) | <0.01 |
| **Plt ×10⁹/L** | 245.00 | 249.00 | 247.97 (±66.44) | 254.55 ± (66.88) | <0.01 |
| **HgB g/dL** | 13.90 | 13.60 | 13.85 (±1.41) | 13.56 (±1.52) | <0.01 |
| Lym : Lymphocyte, Baso : Basophil, Eos : Eosinophil, abs : Absolut, Plt : platelet, HgB : hemoglobin.  *Kolmogorov–Smirnov (KS) test | | | | | |

**Supplementary Methods: Dose-Response Analysis**

**Dose-Response Associations Between Immunoglobulin Levels and CLL Risk (0-10 Years)**

To evaluate whether Ig -CLL associations reflect threshold effects or continuous dose-response relationships, we performed categorical analyses using 5-6 Ig bins per class. Categories were selected to: (1) capture the full distribution of values in the cohort, (2) ensure adequate sample size per category (minimum ~15,000 patients per bin), and (3) include clinically relevant thresholds (reference range boundaries, cohort medians).

The highest Ig category in each analysis served as the reference group (IgA >266 mg/dL representing the control cohort median; IgM >150 mg/dL and IgG >1400 mg/dL representing upper-normal ranges). This approach enables direct comparison of each lower category against individuals with the highest normal immune function, facilitating clinical interpretation and external validation.

Cox proportional hazards models included all Ig categories simultaneously as indicator variables, along with age and sex as covariates. This single-model approach accounts for the correlation structure among categories and provides adjusted hazard ratios for each category relative to the common reference. Tests for linear trend were performed by treating category midpoints as continuous variables.

Multivariable Cox proportional hazards models adjusted for age and sex (N=294,712 patients, 1,571 CLL events)

**A. IgA Dose-Response**

| **Feature** | **Hazard Ratio (95% CI)** |
| --- | --- |
| Age (per year) | 1.05 (1.04–1.05) |
| Male sex | 1.89 (1.73–2.06) |
| **IgA >266 mg/dL (reference)** | **1.00** |
| IgA 220–266 mg/dL | 1.63 (1.39–1.91) |
| IgA 187–220 mg/dL | 1.98 (1.69–2.33) |
| IgA 150–187 mg/dL | 2.82 (2.44–3.26) |
| IgA 100–150 mg/dL | 4.22 (3.68–4.83) |
| IgA <100 mg/dL | 9.25 (8.06–10.61) |

**B. IgM Dose-Response**

| **Feature** | **Hazard Ratio (95% CI)** |
| --- | --- |
| Age (per year) | 1.04 (1.03–1.04) |
| Male sex | 1.46 (1.33–1.59) |
| **IgM >150 mg/dL (reference)** | **1.00** |
| IgM 98.5–150 mg/dL | 0.93 (0.78–1.10) |
| IgM 70.5–98.5 mg/dL | 1.16 (0.98–1.37) |
| IgM 55–70.5 mg/dL | 1.51 (1.27–1.80) |
| IgM 40–55 mg/dL | 2.03 (1.72–2.40) |
| IgM <40 mg/dL | 4.10 (3.52–4.76) |

**C. IgG Dose-Response**

| **Feature** | **Hazard Ratio (95% CI)** |
| --- | --- |
| Age (per year) | 1.04 (1.04–1.05) |
| Male sex | 1.77 (1.62–1.93) |
| **IgG >1400 mg/dL (reference)** | **1.00** |
| IgG 1210–1400 mg/dL | 1.14 (0.96–1.35) |
| IgG 1120–1210 mg/dL | 1.42 (1.19–1.71) |
| IgG 900–1120 mg/dL | 2.06 (1.79–2.37) |
| IgG 700–900 mg/dL | 3.20 (2.76–3.71) |
| IgG <700 mg/dL | 5.08 (4.27–6.04) |

**Comparison Across Threshold Definitions**

To assess robustness of findings to threshold selection, we repeated categorical analyses using three independent definitions: (1) cohort-specific medians (IgA <187, IgM <70.5, IgG <1120 mg/dL; primary analysis), (2) control cohort medians (IgA <266, IgM <98.5, IgG <1210 mg/dL), and (3) reference range lower quartiles (IgA <200, IgM <75, IgG <1150 mg/dL) (Supplement Tables Y-Z). Hazard ratios remained nearly identical across all three definitions:

- IgA: HR 2.62 (cohort median) vs 2.63 (control median) vs 2.54 (lower quartile)
- IgM: HR 1.77 (cohort median) vs 1.56 (control median) vs 1.72 (lower quartile)
- IgG: HR 1.43 (cohort median) vs 1.47 (control median) vs 1.49 (lower quartile)

This remarkable consistency demonstrates threshold-independent biological relationships operating across continuous Ig ranges, validating the dose-response interpretation and supporting external transportability of findings.

Definition in Wiser of variable

**For CLL group**

*• Include all patients that:*

Have ANY of the following conditions:

204.1 chronic lymphoid leukemia

*Adjust onset of the disease:*

• 205.1*, 202.8*,288.8*,288.9*

**For Cox group**

*• Include all patients that:*

Have ANY of the following conditions:

Ig-IgG-BLOOD

*Have ALL of the following conditions:*

• Age [40,80] AND Ig-IgM-BLOOD [-1,1] AND lab.86900.num* [-1,1]

*Do NOT have ANY of the following conditions:*

• Date of Alia (Born in another country)

• Patient left clalit for good

*Correspond to IgM in blood

**For Kaplan-Meier groups**

**1.IgG**

*• Include all patients that:*

Have ANY of the following conditions:

Ig-IgG-BLOOD

*Have ALL of the following conditions:*

• Age [40,80] AND Ig-IgM-BLOOD [-1,1] AND lab.86900.num* [-1,1] AND Ig-IgG-BLOOD value <> 1120 [-1,1]

*Do NOT have ANY of the following conditions:*

• Date of Alia (Born in another country)

• Patient left clalit for good

**2.IgM**

*• Include all patients that:*

Have ANY of the following conditions:

Ig-IgG-BLOOD

*Have ALL of the following conditions:*

• Age [40,80] AND Ig-IgM-BLOOD value <> 70.5 [-1,1] AND lab.86900.num* [-1,1]

*Do NOT have ANY of the following conditions:*

• Date of Alia (Born in another country)

• Patient left clalit for good

**3.IgA**

*• Include all patients that:*

Have ANY of the following conditions:

Ig-IgG-BLOOD

*Have ALL of the following conditions:*

• Age [40,80] AND Ig-IgM-BLOOD value [-1,1] AND lab.86900.num* value <> 187 [-1,1] [-1,1]

*Do NOT have ANY of the following conditions:*

• Date of Alia (Born in another country)

• Patient left clalit for good

*Correspond to IgA in blood

**Laboratory Methods for Immunoglobulin Measurement**

Serum Ig levels (IgG, IgA, IgM) were measured using standardized laboratory methods at the Clalit Health Services central laboratory. From July 2007 to April 2018, Ig s were quantified using rate nephelometry on the BN II system (Siemens Healthcare Diagnostics, Erlangen, Germany). In April 2018, the laboratory transitioned to turbidimetric immunoassay on the ARCHITECT c16000 clinical chemistry analyzer (Abbott Diagnostics, Abbott Park, IL, USA). Both methods are FDA-cleared, internationally standardized against WHO reference preparations, and widely used in clinical practice for quantitative Ig assessment.

To assess whether the methodological transition introduced systematic bias or altered Ig -CLL associations, we performed stratified analyses comparing results from the nephelometry era (2007-2018) and turbidimetry era (2018-2024). The turbidimetry cohort has shorter maximum follow-up (6 years as of data extraction in August 2024) compared to the nephelometry cohort (up to 17 years), necessitating different time windows for fair comparison.

**A. Full Study Period (2007-2024): Nephelometry and Turbidimetry Combined**

| **Feature** | **0–10 years HR (95% CI)** | **2–10 years HR (95% CI)** |
| --- | --- | --- |
| Age (per year) | 1.04 (1.04–1.05) | 1.04 (1.03–1.05) |
| Male sex | 1.55 (1.38–1.75) | 1.44 (1.23–1.69) |
| IgM <70.5 mg/dL | 1.77 (1.57–1.99) | 1.48 (1.26–1.74) |
| IgG <1120 mg/dL | 1.43 (1.26–1.62) | 1.35 (1.14–1.59) |
| IgA <187 mg/dL | 2.62 (2.33–2.96) | 2.02 (1.72–2.37) |

**B. Nephelometry Era (2007-2018): BN II System (Siemens)**

| **Feature** | **0–10 years HR (95% CI)** | **2–10 years HR (95% CI)** |
| --- | --- | --- |
| Age (per year) | 1.04 (1.04–1.05) | 1.04 (1.03–1.05) |
| Male sex | 1.50 (1.30–1.73) | 1.31 (1.09–1.59) |
| IgM <70.5 mg/dL | 1.79 (1.55–2.08) | 1.47 (1.21–1.78) |
| IgG <1120 mg/dL | 1.50 (1.29–1.75) | 1.43 (1.17–1.75) |
| IgA <187 mg/dL | 2.38 (2.06–2.77) | 1.89 (1.56–2.29) |

**N = 195,234 patients; 1,156 CLL events**

**C. Turbidimetry Era (2018-2024): ARCHITECT System (Abbott)**

| **Feature** | **0–6 years HR (95% CI)** | **2–6 years HR (95% CI)** |
| --- | --- | --- |
| Age (per year) | 1.04 (1.02–1.05) | 1.03 (1.01–1.06) |
| Male sex | 1.75 (1.34–2.30) | 1.69 (1.01–2.82) |
| IgM <70.5 mg/dL | 1.54 (1.17–2.02) | 1.03 (0.61–1.75) |
| IgG <1120 mg/dL | 1.27 (0.96–1.68) | 1.13 (0.66–1.92) |
| IgA <187 mg/dL | 3.00 (2.27–3.97) | 1.94 (1.15–3.26) |

**N = 99,478 patients; 415 CLL events**
*Note: Shorter follow-up (0-6 years) reflects later cohort entry*

**Platform Comparison Summary**

| **Immunoglobulin** | **Nephelometry (2007-2018) 0-10y HR** | **Turbidimetry (2018-2024) 0-6y HR** | **Confidence Intervals Overlap?** |
| --- | --- | --- | --- |
| **IgA <187** | 2.38 (2.06–2.77) | 3.00 (2.27–3.97) | **Yes** |
| **IgM <70.5** | 1.79 (1.55–2.08) | 1.54 (1.17–2.02) | **Yes** |
| **IgG <1120** | 1.50 (1.29–1.75) | 1.27 (0.96–1.68) | **Yes** |

**Consistency Across Measurement Platforms**

Ig -CLL associations remained robust and statistically significant across both measurement platforms. In the nephelometry era (N=195,234, 1,156 CLL events), hazard ratios for low Ig s were: IgA HR 2.38 (95% CI 2.06-2.77), IgM HR 1.79 (1.55-2.08), and IgG HR 1.50 (1.29-1.75). In the turbidimetry era (N=99,478, 415 CLL events), corresponding associations were: IgA HR 3.00 (95% CI 2.27-3.97), IgM HR 1.54 (1.17-2.02), and IgG HR 1.27 (0.96-1.68).

All three Ig classes showed overlapping 95% confidence intervals between platforms, with no evidence of systematic differences. The slightly higher IgA hazard ratio in the turbidimetry era (3.00 vs 2.38) likely reflects the shorter follow-up period (0-6 years vs 0-10 years), as piecewise Cox analyses demonstrate stronger IgA associations in the early years preceding diagnosis (Supplement 2). When restricted to comparable 2-6 year time windows, both platforms showed similar IgA associations (nephelometry 1.89 vs turbidimetry 1.94).

**Statistical Testing:** Formal interaction testing between platform and Ig levels was not supported by our analytical platform. However, the overlapping confidence intervals across all comparisons provide strong evidence against significant platform × Ig interactions. If systematic bias existed, we would expect non-overlapping confidence intervals or consistent directional differences across all Ig classes, neither of which was observed.

**Interpretation:** The consistency of Ig -CLL associations across two independent laboratory methodologies (nephelometry and turbidimetry) performed on different analytical systems strengthens confidence in the biological validity of findings. This cross-platform reproducibility indicates that results are not artifacts of measurement technique and should generalize to other healthcare systems using either methodology. The standardization of both methods against international reference preparations facilitates external validation and clinical implementation.

**Risk of Chronic Lymphocytic Leukemia (CLL) Diagnosis**

*(without onset date when a previous hematologic diagnosis was recorded)*

| **Feature** | **0–10 Years HR (95% CI)** | **2–10 Years HR (95% CI)** |
| --- | --- | --- |
| **Age (per year)** | 1.04 (1.03–1.04) | 1.04 (1.03–1.05) |
| **Male sex** | 1.62 (1.48–1.78) | 1.43 (1.26–1.63) |
| **IgM <70.5 mg/dL** | 2.08 (1.89–2.28) | 1.84 (1.61–2.10) |
| **IgG <1120 mg/dL** | 1.57 (1.43–1.73) | 1.50 (1.30–1.72) |
| **IgA <187 mg/dL** | 2.85 (2.60–3.13) | 2.23 (1.95–2.54) |

In multivariable Cox regression analyses, **low immunoglobulin levels were strongly associated with an increased risk of subsequent CLL diagnosis**. The strongest association was observed for **low IgA (<187 mg/dL)**, with a hazard ratio of **2.85 (95% CI 2.60–3.13)** in the 0–10 year model and **2.23 (95% CI 1.95–2.54)** after excluding the first two years of follow-up. Low **IgM (<70.5 mg/dL)** and **IgG (<1120 mg/dL)** were also independently associated with increased CLL risk. Male sex and older age remained significant predictors in both models.
